# Gut microbiota interplay with autophagy-EMT dynamics in colorectal cancer

**DOI:** 10.3389/fcell.2025.1608248

**Published:** 2025-08-21

**Authors:** Tiziana Vescovo, Giulio Bontempi, Mohammadreza Bayat, Lucia Piredda, Marco Fidaleo, Raffaele Strippoli, Manuela Antonioli

**Affiliations:** ^1^ Department of Epidemiology, Preclinical Research and Advanced Diagnostics, National Institute for Infectious Diseases IRCCS “L. Spallanzani”, Rome, Italy; ^2^ Department of Molecular Medicine, University of Rome “Sapienza”, Rome, Italy; ^3^ PhD Program in Cellular and Molecular Biology, Department of Biology, University of Rome “Tor Vergata”, Rome, Italy; ^4^ Department of Biology and Biotechnologies “Charles Darwin”, Sapienza University of Rome, Rome, Italy; ^5^ Research Center for Nanotechnology Applied to Engineering (CNIS), Sapienza University of Rome, Rome, Italy; ^6^ Department of Biology, University of Rome “Tor Vergata”, Rome, Italy

**Keywords:** microbiota, autophagy, epithelial mesenchymal transition, colorectal cancer (CRC), diagnosis, prognosis, therapeutic intervention

## Abstract

The human microbiota is composed of a complex community of microorganisms essential for maintaining host homeostasis, especially in the gastrointestinal tract. Emerging evidence suggests that dysbiosis is linked to various cancers, including colorectal cancer (CRC). The microbiota contributes to CRC development and progression by influencing inflammation, genotoxic stress, and key cell growth, proliferation, and differentiation pathways. Certain bacterial species, including *Fusobacterium nucleatum* and *Escherichia coli*, play a role in tumorigenesis by facilitating epithelial-mesenchymal transition (EMT), perturbing autophagy, and supporting immune evasion. In contrast, beneficial microorganisms such as *Bifidobacterium* and *Lactobacillus* provide protective effects by boosting immune surveillance and supporting the integrity of the intestinal barrier. This review examines the complex connection between gut microbiota and CRC, emphasizing how changes in microbial composition facilitate tumor development and influence treatment outcomes. We cover recent progress in microbiota-based biomarkers for CRC diagnosis and prognosis, showcasing their promise for early detection and improved patient stratification. Furthermore, we explore microbiota-focused therapeutic methods such as probiotics, prebiotics, faecal microbiota transplantation (FMT), and precision antibiotics, which show potential to complement standard CRC treatments. By highlighting the latest advancements in this area, we emphasise how microbiome research is transforming our comprehension of CRC and leading to new diagnostic and treatment approaches.

## 1 Introduction

Colorectal cancer (CRC) is one of the most prevalent malignancies worldwide and a leading cause of cancer-related mortality (Morgan et al., 2023). Its pathogenesis is complex, involving genetic, epigenetic, and environmental factors, including chronic inflammation and microbial dysbiosis (Li et al., 2024). A key process implicated in CRC progression and metastasis is epithelial-to-mesenchymal transition (EMT), during which epithelial cells acquire mesenchymal traits, enhancing motility and invasiveness (Lu et al., 2023). Also, autophagy, a lysosome-mediated degradation pathway, plays a dual role in CRC by modulating tumor initiation, survival, and therapy resistance (Hu et al., 2021). Recent advances have shed light on the gut microbiota as a pivotal regulator of intestinal homeostasis by influencing various physiological processes, including nutrient absorption, immune modulation, and protection against pathogens (Jyoti and Dey, 2025). Emerging evidence reveals that microbial communities also interact with molecular pathways such as EMT and autophagy, contributing to CRC initiation and progression (Li et al., 2024). This review explores the multifaceted crosstalk between gut microbiota, EMT, and autophagy in the context of CRC, aiming to elucidate their interdependent roles in tumor development and therapeutic responses.

## 2 Mechanism of microbiota- tumor interactions

Initial hypotheses on cancer development proposed that pathogens were the leading causes. However, only a small number of tumours were directly associated with viral infections (e.g., Epstein-Barr, human papillomavirus, and hepatitis viruses), and somatic DNA mutations subsequently assumed a pivotal role in tumour onset. Only recently, microorganisms have acquired new attention in cancer research, with the microbiota extensively investigated in this context. The microbiota comprises several microorganisms, including bacteria, archaea, fungi, protozoa, and viruses. These are central in digestion, immune regulation, and protection against pathogens, and their assortment depends on several factors like diet, lifestyle, genetic factors, and the environment. Recently, the advancements in genome sequencing allowed the deep characterisation of microbiota compositions, thus letting to evaluate its link to several diseases, like cancer (Wei et al., 2021; van Vorstenbosch et al., 2023). However, understanding the role of the microbiota in cancer development is multifaceted and questioned, mainly due to the complex nature of the interactions between microorganisms and the host. While some microorganisms have been linked to head-neck, pancreatic, and colorectal cancers, their precise role is still uncertain and highly debated. Indeed, some phyla may contribute to tumorigenesis, while others assist in maintaining a healthy microenvironment, thereby supporting cancer prevention and enhancing the effectiveness of anti-cancer therapies (Goodman and Gardner, 2018). Microbiota colonises various epithelial surfaces (Klaassen and Cui, 2015), like the skin, the oral cavity and the respiratory and urogenital tracts; nevertheless, the gut-associated microorganisms are mainly characterised. The most prevalent phyla of the gut microbiota are represented by Firmicutes and Bacteroidetes, alongside smaller populations of Proteobacteria and Actinobacteria (Goodman and Gardner, 2018; Procházková et al., 2023). Given that the composition of microbiota differs among individuals, there is no universally defined “healthy” microbiome; instead, researchers categorise microbial community profiles into “enterotypes” (Costea et al., 2017).

To date, several molecular, computational, and imaging techniques can support the characterisation of the microorganisms’ diversity and function across different cancers. For instance, next-generation sequencing (NGS) is one of the most widely used tools for microbiome analysis, allowing the identification of microorganisms in environments previously considered sterile. The microbiome has been reported for more than 30 cancer types, opening up to a novel paradigm shift where the microbiome equilibrium could potentially co-participate in various cancer-related processes (Azevedo et al., 2020). Therefore, metagenomics studies, including both the analysis of 16S rRNA variability and the shotgun metagenomic sequencing, enable species-level resolution and can detect oncogenic bacterial genes, such as colibactin-producing *Escherichia coli,* which has been associated with CRC (Mäklin et al., 2024). Quantitative PCR (qPCR) is an effective technique for detecting specific cancer-associated microbes with high sensitivity. It has been extensively used to identify and quantify *Helicobacter pylori* in gastric cancer, proving its strong correlation with tumor development (Castaneda et al., 2020). In addition, fluorescence *in situ* hybridization (FISH) is a valuable microscopy-based technique that allows direct visualization of bacteria within tumor tissues. Very recently, this approach has been applied to confirm the spatial presence of *Fusobacterium nucleatum* in colorectal tumors, supporting its role in tumor onset (Wang et al., 2025). Finally, other omics approaches, which include metabolomics and proteomics, provide insights into microbial-derived metabolites that influence cancer biology (Zhang et al., 2023; Gou et al., 2024). Of relevance, germ-free mouse models have provided crucial *in vivo* evidence of microbial contributions to tumorigenesis (Jans and Vereecke, 2024). Altogether, the integration of these techniques is essential for comprehensively understanding the tumor microbiome and advancing microbiome-targeted cancer therapies, while future research should refine detection methods to increase microbial diagnostics and interventions.

A proper assortment of the human microbiota is essential for maintaining the healthy physiology of the host, as it impacts immune homeostasis, metabolism, and inflammation. Increasing evidence suggests that the microbiota composition, like host-specific factors, can influence tumorigenesis by altering this equilibrium, thus promoting or inhibiting cancer development (Jiang et al., 2024). The microbiota contributes to tumor onset and progression, sustaining chronic inflammation, genotoxic stress, and epigenetic modifications. In oral squamous cell carcinoma (OSCC), seven bacterial strains have been identified within the tumour microenvironment (Cai et al., 2024), with *F. nucleatum* promoting the expression of the *SNAI2* gene and the subsequent epithelial-mesenchymal transition (EMT). Consistent with this, Mi Ra Yu et al. found that *F. nucleatum* increased EMT-associated transcription factors such as SNAIL and SLUG and decreased E-cadherin in LOVO cells in a dose-dependent manner, whereas *P. gingivalis* infection did not affect EMT-associated molecules (Yu et al., 2020). Besides *F. nucleatum,* colibactin-producing *E. coli* (CoPEC) induces DNA double-strand breaks, DNA mutations, genomic instability, and cellular senescence. Infected cells produce a senescence-associated secretory phenotype (SASP), which is involved in the increase in tumorigenesis observed in CRC mouse models infected with CoPEC. This finding correlated with the induction of EMT, which led to the emergence of cells exhibiting Cancer Stem Cell (CSC) features. Overall, CoPEC might worsen CRCs by promoting the development of cancer stem cells that are highly resistant to chemotherapy (Dalmasso et al., 2024). While several information have emerged on the functional effect of bacterial toxins in inducing EMT-related features, the molecular mechanisms underlying this have not been further dissected.

Similarly, in gastric cancer, chronic gastritis caused by *H. pylori* has been correlated to DNA damage through reactive oxygen species (ROS) production and ultimately to carcinogenic transformation (Sah et al., 2023). Beyond the gastrointestinal tract, and specifically in pancreatic cancer, oral pathogens such as *P. gingivalis* and *A. actinomycetemcomitans* alter immune responses and promote an inflammatory tumor microenvironment (Pourali et al., 2024). Meanwhile, differently from other tumors, Anaerococcus, *Caulobacter*, and *Streptococcus* are absent in breast cancer tissues, while *Propionibacterium* and *Staphylococcus* are reduced and negatively linked to oncogenic immune features. In this context, *Streptococcus* and *Propionibacterium* positively correlate with T-cell activation genes (Tzeng et al., 2021).

As previously mentioned, the microbiota profoundly impacts the immune system, influencing both innate and adaptive immunity and reinforcing its dual role in cancer. Indeed, some microorganisms show pro-inflammatory properties and facilitate tumorigenesis. For instance, *F. nucleatum* promotes the progression of colorectal cancer by recruiting myeloid-derived suppressor cells (MDSCs) and by inhibiting the cytotoxic T-cell and Natural killer activity (Dadgar-Zankbar et al., 2024). By contrast, balanced microbiota composition is essential for effective anti-tumor immune surveillance. It has been reported that beneficial commensal bacteria such as *Bifidobacterium* and *Lactobacillus* enhance anti-tumor immunity by promoting dendritic cell maturation and cytotoxic T-cell activation (Chen et al., 2022). In addition, it is emerging how the gut microbiota composition significantly influences the immune checkpoint inhibitors efficacy, with specific microbial strains able to boost the immunotherapy response (Jiang and Zhang, 2024).

Furthermore, interactions between the microbiota and tumors go beyond the modulation of the immune response; they also cause metabolic changes that influence tumor growth and viability. It has been reported that certain microorganisms enhance glycolysis and lipid metabolism, providing energy sources which increase tumor growth (Chen et al., 2023). For instance, *Bacteroides uniformis* has a high glycolytic capability and increases butyrate levels, which in turn accumulates in tumor cells, promoting histone deacetylation, apoptosis induction and inhibition of cell proliferation in CRC (Donohoe et al., 2014; Benítez-Páez et al., 2017). Moreover, also amino acid metabolism is influenced, with the administration of *Lactobacillus* and *Bifidobacterium* probiotics increasing plasma tryptophan levels, serotonin synthesis and, in turn, perturbing the tryptophan metabolism (Hou et al., 2023). Intriguingly, the microbiota has been described as modulating hormone metabolism; for instance, in breast cancer, the microbial β-glucuronidase (GUS) enzyme may increase estrogen bioavailability, thus influencing tumor progression (Arnone and Cook, 2022).

In addition, microbiota-derived metabolites also play a dual role in cancer development, acting as both pro- and anti-tumorigenic agents. In this regard, microbiome-derived short-chain fatty acids (SCFAs) levels in faeces have been linked with a higher risk of developing inflammatory diseases and certain cancers (e.g., breast and stomach cancer) (Qu et al., 2023). By contrast, hydrogen sulfide (H2S) produced by *Desulfovibrio* species (Singh et al., 2023) has also been correlated to CRC by inducing DNA damage and altering mitochondrial metabolism (Munteanu et al., 2023). Similarly, N-Nitrosamines produced by *E. coli* and *Clostridium* have been described to promote carcinogenic transformation (Luo et al., 2022). In this regard, drug metabolism and chemoresistance are also altered; for instance, *F. nucleatum* reduces chemotherapy efficacy by modulating autophagy pathways (Liu et al., 2020) and participating in 5-FU resistance in CRC patients (Huang et al., 2022). Therefore, understanding how specific metabolites are involved in cancer may be essential for the development of new therapies specifically targeting the microbiome, as well as for identifying microbial biomarkers to enhance cancer prevention and treatment, in particular for chemo-resistant patients.

## 3 Microbiota and colorectal cancer

Colorectal cancer (CRC) is the second most deadly tumor, with an incidence of 1.84 cases and 0.8 deaths per million worldwide, and it accounted for 9.6% of all diagnosed cancers in 2022 (Xi and Xu, 2021). In early-stage CRC-diagnosed patients, surgical resection is the primary therapeutical approach, often supported by chemo-radiotherapy. However, the efficacy of used treatments may be reduced by frequent drug resistance events, which usually lead to cancer recurrence. In light of these considerations, learning cellular processes involved in CRC development and drug resistance would sustain the identification of new therapeutical strategies for CRC management, which is currently one of the major global public health challenges. CRC is a multifactorial disease influenced by chronic gut inflammation (e.g., inflammatory bowel disease, IBD), intestinal microbiota alteration, immune dysfunctions, genetic mutations, and epigenetic changes of intestinal epithelial cells (IECs). All these factors influence several pathways in the IECs and their microenvironment, which may contribute to CRC pathogenesis.

The links between specific gut microbiota and CRC initiation and progression have been extensively investigated recently (Table 1). To date, few microorganisms in the gut microbiota have been directly linked to CRC development. For example, certain strains of *E. coli* produce a toxin called colibactin, which causes DNA mutations through alkylation, contributing to cancer development (Wilson et al., 2019); meanwhile, *C. rjejuni* releases the cytolethal distending toxin (CDT), a genotoxin with DNAse activity inducing dsDNA breaks (He et al., 2019). Another carcinogenic mechanism, directly dependent on microorganisms, involves the alteration of E-cadherin/Wnt/b-Catenin signaling pathway, which in turn regulates cell proliferation, differentiation, apoptosis, and motility. For instance, the cell-surface adhesin, FadA from *F. nucleatum*, as well as AvrA from some *Salmonella* strains, impact E-cadherin/Wnt/b-Catenin pathway promoting enterocyte translocation during intestinal epithelial invasion (Wu et al., 2012; Rubinstein et al., 2013; Silva-García et al., 2019). Another mechanism affected by the interplay between a somatic host mutation and the activity of a microorganism is represented by specific p53 mutant variants, which exhibit diverse effects depending on the gastrointestinal tract in mice with *Csnk1a1* deletion or Apc^Min^ mutation. It has been reported that p53 mutations show more oncogenic tendencies in the distal gut compared to the proximal gut, where they exhibit a tumor-suppressive function. Notably, this tumor inhibitory function is reversed by the gallic acid polyphenol, a metabolite produced by the microbiota (Kadosh et al., 2020). Therefore, while other observations suggest a protective role of the microbiota from CRC onset, it has also been described that the dysbiosis associated with CRC disrupts the equilibrium between microbic populations and immune cells, promoting inflammation and cancer progression (Chen et al., 2017). In this regard, *F. nucleatum* populates CRC malignant tissues with its DNA sequences and cultivable microorganisms identified in tumor-derived samples. However, the direct association of *F. nucleatum* with CRC development is complicated by the pathobiont nature of this microorganism. While it is typically present in the human body without causing disease or disorders, it can become pathogenic or contribute to cancer when conditions in the host are altered or weakened (Jochum and Stecher, 2020). Moreover, evidence suggests that *F. nucleatum* contributes to the chemoresistance of CRC cells and predicts lower survival rates for patients (Yu et al., 2017). Interestingly, its presence has been detected and cultured from distant metastatic lesions of CRC and in colorectal cancer stem cells (CR-CSCs) (Bullman et al., 2017), as well as been reported in cancer recurrence following surgical and chemotherapeutic interventions. In addition, *F. nucleatum* can elicit innate immune responses in CR-CSCs, thus suggesting a possible association between the bacterium, metastasis, and the high relapse percentage in CRC (Cavallucci et al., 2022).

**TABLE 1:** The role of gut microbiota in colorectal cancer.

Microorganism	Scientific evidence	Role in CRC	Ref.
*E.coli* (CoPEC)	Produces colibactin toxin, causing DNA mutations through alkylation	Contributes to cancer development	Costea et al. (2017)
*C. jejuni*	Releases cytolethal distending toxin (CDT), a genotoxin with DNAse activity inducing dsDNA breaks	Contributes to cancer development	He et al. (2019)
*F. nucleatum*	Cell-surface adhesin, FadA, impacts the E-cadherin/Wnt/b-Catenin signaling pathway	Promotes enterocyte translocation during intestinal epithelial invasion	Rubinstein et al. (2013); Silva-García et al. (2019)
*F. nucleatum*	Populates CRC malignant tissues with its DNA sequences and cultivable microorganisms identified in tumor-derived samples	Complex associations with CRC development contribute to the chemoresistance of CRC cells and predict lower survival rates present in distant metastatic lesions of CRC and CRC stem cells	Bullman et al. (2017); Chen et al. (2017); Yu et al. (2017); Jochum and Stecher (2020)
*Salmonella* spp.	AvrA impacts E-cadherin/Wnt/b-Catenin signaling pathway	Promotes enterocyte translocation during intestinal epithelial invasion	Wu et al. (2020)

**FIGURE 1:**
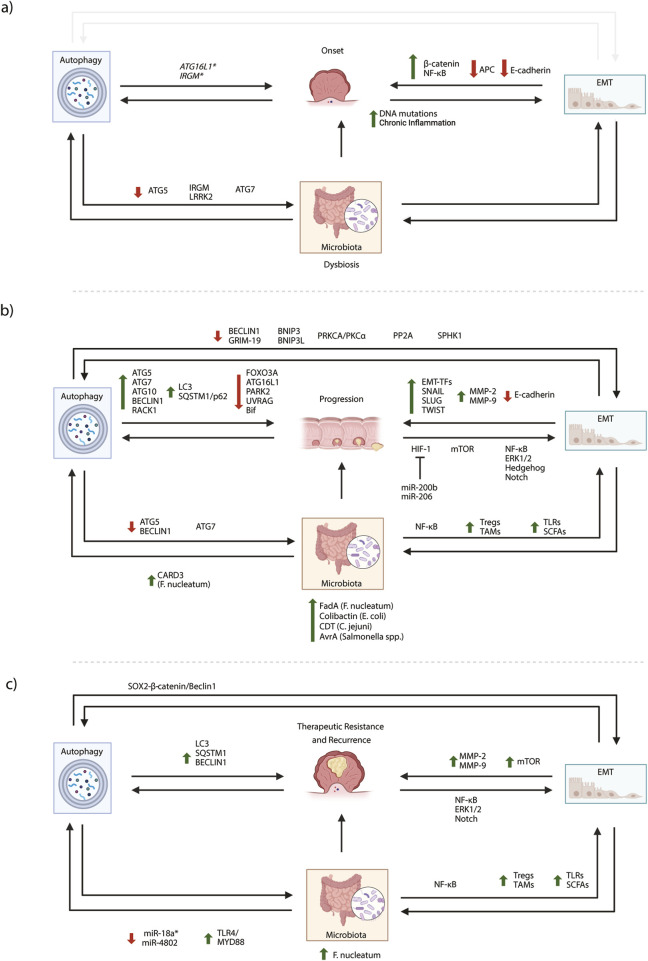
Microbiota, autophagy and EMT axis in CRC pathology. Changes in microbiota composition contribute to CRC development and progression by influencing inflammation, facilitating EMT and perturbing autophagy. Viceversa autophagy or EMT dysregulation could induce intestinal dysbiosis promoting CRC. According to this crosstalk between microbiota autophagy and EMT, the role of main proteins, molecules and pathways related to colorectal cancer onset **(a)**, progression **(b)**, therapy resistance and cancer recurrence **(c)** have been schematically reported. Red arrows: downregulation, green arrows: upregulation. Created in BioRender. Antonioli, M. (2025) https://BioRender.com/gvv1dnm.

The gut microbiota modifies several cellular mechanisms involved in CRC, including inflammation, DNA damage, endoplasmic reticulum (ER) stress, autophagy, and EMT. All these mechanisms *per se* are associated with CRC initiation, progression, and therapy response; only more recently, the microbiota arose as modulating them in CRC tumorigenesis (Zhao et al., 2023; Figures 1a–c). Chronic inflammation is one of the more extensively described alterations influenced by the gut microbiota in CRC. It has been reported that *F. nucleatum and Bacteroides fragilis*, trigger immune responses, leading to increased production of pro-inflammatory cytokines like IL-6, IL-17, and TNF-α (Park et al., 2018). As mentioned, gut microbiota also contributes to DNA damage and genomic instability, with genotoxins produced by *Clostridium* genera, *M. morganii* and colibactin-producing *E. coli* described in DNA damage and, therefore CRC development. Furthermore, microbiota and their toxins or metabolites can induce prolonged ER stress and unfolded protein response (UPR) signalling in the gut, with serious implications for intestinal inflammation and cancer development (Di Mattia et al., 2025). In recent years, autophagy and EMT have been increasingly recognized as important cellular mechanisms influenced by the gut microbiota in colorectal cancer (CRC).

## 4 Autophagy in CRC onset and progression

Among cellular mechanisms involved in the crosstalk between the gut microbiota and the intestinal epithelial cells (IECs), autophagy has been extensively described in CRC development and progression. Autophagy is a catabolic process degrading intracellular components through lysosome fusion (Mizushima et al., 2011). Different types of selective autophagy exist and take part in the quality control of cells (Johansen and Lamark, 2011), such as the selective degradation of microorganisms and viruses (i.e., xenophagy), which attempts to remove invading pathogens, ensuring and remodeling the composition of the gut microbiota (Klionsky et al., 2016; Larabi et al., 2020). Autophagy alterations are involved in several human diseases. In cancer, it plays a controversial role, acting as both a tumor suppressor and a tumor-promoting mechanism (Klionsky et al., 2021). On the one hand, it can inhibit cancer onset by maintaining cellular balance and enhancing immune surveillance. On the other hand, it provides energy for tumor growth and contributes to chemoresistance. Moreover, autophagy also contributes to stemness maintenance, thus promoting tumor recurrence (Nazio et al., 2019). Specifically in the gastrointestinal tract, autophagy ensures homeostasis, integrity, and repair, mainly supporting intestinal barrier function in response to stress (Foerster et al., 2022). The intestinal barrier is a semi-permeable structure that facilitates nutrient absorption, immune sensing, and protection from harmful substances and pathogens. The first defence is the monolayer of intestinal epithelial cells (IECs) replenished by intestinal stem cells (ISCs) through continuous turnover (Vancamelbeke and Vermeire, 2017). Autophagy regulates the proliferation and regeneration of ISCs and secretory cells, with significant implications for CRC. Below the epithelial layer, immune cells from lamina propria (e.g., T cells, B cells, macrophages, and dendritic cells) support bowel immune surveillance. Dysfunctions or damage of this barrier lead to several intestinal pathologies, with inflammatory bowel disease (IBD) as a high-risk factor for CRC onset (Stidham and Higgins, 2018). In this context, the role of autophagy in IBD has recently gained prominence (Shao et al., 2021), with polymorphisms of ATG16L1 and IRGM described as risk factors for Crohn’s disease. Interestingly, it has been shown that NOD2 induces autophagy to remove intracellular pathogens by directly interacting with ATG16L1 (Fritz et al., 2011). Moreover, IRGM and LRRK2 are autophagic genes involved in bacterial infection response and the pathogenesis of IBD, which may contribute to CRC development (Liu and Lenardo, 2012). The role of autophagy in CRC development and progression is still widely debated due to its dualist nature in cancers and the complexity of different CRC features. Approximately 75% of CRCs are sporadic and mostly caused by the loss of the adenomatous polyposis coli (APC) gene (Guinney et al., 2015), followed by p53 and KRAS mutations, thus causing spontaneous tumor development and progression. Similarly to other Ras-driven cancers, CRC also presents enhanced autophagy (Guo et al., 2011). However, ATG5 has been reported to be downregulated in 95% of CRC cases, although its expression increases with invasion into lymphovascular tissue (Cho et al., 2012); also, ATG10 upregulation correlates with tumor lymph node metastasis and invasion in CRC tissues (Jo et al., 2012). Differently, in a CRC mouse model, Atg7 deficiency of IECs prevents tumor initiation through a microbiome-influenced immune response and suppresses tumor growth (Lévy et al., 2015). Moreover, CRC patients with the ATG16L1 T300A genetic variant have a longer life expectancy than the WT counterpart, with higher production of type I IFN via the MAVS pathway, which may help constrain CRC through immune invasion (Grimm et al., 2016). Interestingly, both the autophagic markers LC3 and SQSTM1 are associated with the prognosis of CRC (Niklaus et al., 2017). Indeed, low LC3 levels correlate with increased treatment response and higher overall survival of patients with advanced CRC (Yang et al., 2015). Another crucial protein involved in autophagy induction, BECLIN-1, has been described in CRC development despite its controversial role. Indeed, high BECLIN-1 levels correlate both with extended survival of CRC patients and with chemoresistance depending on cancer stages (Li et al., 2009; Park et al., 2013). Furthermore, a meta-analysis study has highlighted that overexpression of BECLIN-1 is associated with a poor prognosis and metastasis occurrence in CRC patients (Han et al., 2014). Other components of the BECLIN-1 complex have been implicated in CRC; for instance, UVRAG is heterozygous mutated in CRC, while Bif-1 is poorly expressed (Coppola et al., 2008). Over autophagy initiation, selective autophagy has been described in CRC, with mitophagy playing a dualistic role. On the one hand, mitophagy inhibition impairs tumor growth in both sporadic and colitis-associated cancer models (Devenport et al., 2021). On the other, its activation stimulates the presentation of MHC class I on the cell surface, triggering an anti-tumor immune response by induction on CD8+T-cells increases in intestinal cancer (Ziegler et al., 2018). Notably, the mitophagy-related protein PARK2 has also been described as haploinsufficient in 33% of CRCs (Poulogiannis et al., 2010). Moreover, the kinase RACK1 plays a key role in colonic epithelial carcinogenesis, and its expression gradually increases, positively correlating with tumor aggressiveness and, inversely, with patient survival. RACK1 supports tumor development by inducing autophagy in colon cancer cells, promoting proliferation, and inhibiting apoptosis (Xiao et al., 2018). In addition, autophagy regulates the degradation of FOXO3A, a transcription factor involved in apoptosis. Impairment of autophagy increases FOXO3A levels, sensitizing cancer cells to cell death (Fitzwalter et al., 2018). Finally, autophagy inhibition enhances apoptosis in colon cancer cells, activating p53 and unfolded protein response (UPR) with anticancer effect (Sakitani et al., 2015). Altogether, this evidence suggests a fine and multifaceted modulation of autophagy in CRC pathology, which has the potential to unveil novel biomarkers and/or therapeutic targets. The involvement of the described autophagy-related proteins in CRC development is graphically depicted in Figures 1a–c. The description of autophagy’s role in CRC is summarised in Table 2.

**TABLE 2:** The role of autophagy in colorectal cancer.

Autophagic factors	Scientific evidence	Role in CRC	Ref.
ATG5	Increased expression in patients	CRC invasion	Yang et al. (2018)
	Atg5 deficiency in mice	Gut microenvironment alteration (CRC onset)	Cho et al. (2012)
Atg7	Atg7 deficiency in mice	Reduced tumor initiation and growth	Lévy et al. (2015)
ATG10	ATG10 overexpression in patients	Association with CRC invasion and metastasis	Jo et al. (2012)
ATG16L1	T300A genetic variant in CRC patients	Increased immune response and patients survival	Grimm et al. (2016)
	T300A variant: defective autophagy in mice	Risk factors for Crohn’s disease and CRC onset	Grimm et al. (2016)
BECLIN-1	Overexpressed in CRC patients	Poor prognosis and metastasis formation	Park et al. (2013)
	High levels in metastatic CRC patients treated with 5-FU-based adjuvant therapy	correlate with chemoresistance and poor survival Pro-Tumor	Park et al. (2013)
	High expression	Increased survival of advanced-stage CRC patient	Han et al. (2014)
LC3	Low levels correlate with patients with advanced CRC	Increased responses to treatment and survival	Park et al. (2013); Niklaus et al. (2017)
RACK1	Induced autophagy in CRC cells	CRC onset	Xiao et al. (2018)
UVRAG	Monoallelically mutated at high frequency in human CRCs	Suppressed proliferation and tumorigenicity in CRC cells	Liang et al. (2006)
Bif-1	Low expression in CRC patients	CRC onset	Coppola et al. (2008)
PARK2	Described as haploinsufficient in 33% of CRCs	Tumor suppressor in CRC	Poulogiannis et al. (2010)

### 4.1 Autophagy and gut microbiota in CRC

Autophagy is crucial in regulating the gut microbiota and immune function. In this regard, autophagy dysregulation is associated with intestinal dysbiosis, impaired intracellular bacterial clearance, and amplified intestinal inflammation (Larabi et al., 2020), all processes extensively linked to CRC onset. It has been described that Atg5 knockout mice of intestinal epithelium show a relevant alteration of gut microbiota associated with persistent immune response, intestinal inflammation, and IBD occurrence (Yang et al., 2018). On the other hand, Atg7 deficiency in a mouse model of sporadic colorectal cancer (Apc model) leads to intestinal dysbiosis and infiltration of antitumor immune cells, thus decreasing tumor burden (Lévy et al., 2015). Authors described that autophagy impairment has anti-cancer functions by regulating the microbiota, with antibiotics reducing the anti-tumoral response and promoting tumoral foci formation (Lévy et al., 2015).

By contrast, it has been reported that several microorganisms of the gut microbiota (e.g., *F. nucleatum, Campylobacter Campylobacter jejuni, E.* and *Salmonella*) modulate autophagy at different levels (Xue and Zhu, 2018; Su et al., 2020; Wu et al., 2020; Fukushima et al., 2022; Liu et al., 2022), thus opening to the possibility that microbiota composition could influence autophagy of intestinal cells. Notably, as mentioned above, gut microbiota triggers ER stress, which can then induce autophagy through various related pathways (i.e., UPR, Akt signalling), to degrade misfolded proteins and reduce cellular stress. In this context, autophagy dysregulation is linked to unresolved ER stress, leading to pro-inflammatory signalling activation and CRC development (Beilankouhi et al., 2023; He et al., 2025).

In IECs, infection with COPEC stimulates autophagy, exerting anti-cancer properties protecting from inflammation, DNA damage, and cell proliferation induced by bacteria. Differently, in high COPEC presence, Atg16L1 deficiency leads to an increased number and size of CRC masses compared to the autophagy-sufficient counterpart (Salesse et al., 2021). It has been described that the colibactin-producing *E. coli* (COPEC) mainly colonizes the colon mucosa of CRC patients and increases carcinogenesis in CRC-susceptible mouse models (Lucas et al., 2020). Interestingly, intestinal cancer cells infected with *F. nucleatum* show increased autophagy both *in vivo* and *in vitro*, linked to CRC metastatization and chemoresistance (Yu et al., 2017; Chen et al., 2020). In this context, higher expression of CARD3 has been observed, along with positive regulation of autophagy and increased expression of proteins related to CRC migration, invasion, and metastasis. CARD3 downregulation or chloroquine treatment slows metastasis triggered by bacteria, suggesting that the microbiota could regulates CRC progression through autophagy (Chen et al., 2020). Moreover, in CRC patients, post-chemotherapy high *F. nucleatum* concentration promotes chemo-resistance to 5-FU and oxaliplatin by inducing autophagy via the TLR4/MYD88 signalling pathway, downregulating specific miRNAs miR-18a* and miR-4802 (Cheng et al., 2020). Interestingly, the expression of BECLIN-1 inversely correlates with the quantity of *F. nucleatum* DNA in CRC tissue, suggesting that autophagy may contribute to its removal from the tumor microenvironment (Haruki et al., 2020). Overall, autophagy impairment in the bowel can imbalance the gut microbiota, leading to inflammation and cancer. The capability of autophagy to maintain a proper equilibrium for a healthy microbiota influences immune responses and gut cell functions, thus impacting on CRC development and progression. Therefore, a better knowledge of the intricated relationship between autophagy and the gut microbiota could offer new prevention and treatment strategies for CRC. The autophagy-microbiota interplay in CRC is schematically depicted in Figure 1, panels a, b and c.

## 5 Epithelial-mesenchymal transition in colorectal cancer

### 5.1 EMT in CRC progression

In the progression of CRC, the ability of epithelial cells to acquire migratory and invasive properties is sustained by the Epithelial-mesenchymal transition (EMT). EMT promotes tumor invasion, metastasis, and resistance to apoptosis and chemotherapy. The resulting mesenchymal cells can invade surrounding tissues, survive in circulation, and establish new colonies at distant sites through a reverse process called mesenchymal-epithelial transition (MET) (Takiishi et al., 2017). In colorectal cancer, many cellular pathways have been described in the transdifferentiation that occurs during EMT. Almost all of them regulate the expression of transcription factors (EMT-TFs), such as SNAIL, SLUG, and TWIST, reducing E-cadherin levels and promoting the expression of several mesenchymal markers. In particular, the TGF-β/SMAD (Wang et al., 2021; Wu N. et al., 2021), the Wnt/β-catenin (Bian et al., 2020), the NF-κB (Jurjus et al., 2016; Kapoor and Padwad, 2023) and the NOTCH (Walter et al., 2020) pathways modulate the CRC microenvironment through cytokines, growth factors, and the reorganization of extracellular matrix (ECM) components. Interestingly, NF-κB, metalloproteinases (MMPs), and NOTCH alterations have been described in CRC, further contributing to treatment failure associated with chemo-resistance (Zheng et al., 2015). Similarly, the ERK pathway modulates cell adhesion by downregulating E-cadherin and upregulating MMPs (e.g., MMP-2 and MMP-9) (Wang et al., 2017). In addition, the Hedgehog (Hh) pathway, crucial in embryonic development and tissue homeostasis, has been implicated in the pathogenesis of CRC since it is aberrantly activated, promoting the expression of several EMT genes (Centelles, 2012; Magistri et al., 2018; Guo et al., 2021). Under hypoxic conditions, the HIF pathway directly or indirectly activates EMT target genes in CRC (Shang et al., 2017) promoting angiogenesis and ECM remodeling through the induction of angiogenic factors like VEGF, thus supporting new blood vessel formation (Xu et al., 2018). In colorectal cancer (CRC), the mTOR pathway is crucial for cell growth, metabolism, and protein synthesis. Its dysregulation promotes EMT in different CRC subtypes by influencing EMT-TFs expression, as well as modulating E-cadherin and other proteins involved in cell adhesion and cytoskeletal reorganization (Liao et al., 2022). Like others, also the mTOR pathway interconnects with other pathways related to EMT (e.g., TGF-β/Smad and Wnt/β-catenin) (Wu et al., 2023), as well as with autophagy. The involvement of EMT and its related pathways in CRC development, is graphically depicted in Figures 1a–c. The description of EMT-related pathways role in CRC is listed in Table 3.

**TABLE 3:** The role of EMT-related pathways in colorectal cancer.

Pathways	Altered mechanism	Role in CRC	Ref.
TGF-β/SMAD	Aberrant activation by LINC00941 upregulation and/or HAPLN1 reduction	Principal EMT activator. Crosstalk with other pathways (e.g., Wnt/β-catenin)	Wang et al. (2021); Wu et al. (2021b)
NF-κB	Increased production of inflammatory and anti-apoptotic mediators	Links inflammation to cancer development	Jurjus et al. (2016)
Wnt/β-catenin	APC inactivation and specific transcriptional signature induction	Onset and progression	Bian et al. (2020)
Notch	Increased activation by altered MMP9 activity	Modulation of tumor microenvironment	Walter et al. (2020)
ERK pathway	Activation of EMT-inducing transcription factors; regulation cell adhesion molecules and MMPs	Progression and invasion	Wang et al. (2017); Xu et al. (2018); Ni et al. (2023)
Hedgehog	Positively regulated by increased GLI1 and POU4F2 expression	EMT genes expression and dedifferentiation	Centelles (2012); Guo et al. (2021)
HIF	Increased Ascl2 expression (blocked by miR-200b); inhibition by miR-206; induction of angiogenic factors like VEGF	Angiogenesis and progression	Shang et al. (2017); Xu et al. (2018)
PI3K/Akt/mTOR	Regulation of EMT-related transcription factors; protein translation and synthesis; crosstalk with autophagy	Cell growth, angiogenesis and metastasis	Liao et al. (2022); Wu et al. (2023)

### 5.2 EMT and gut microbiota in CRC

Besides autophagy, EMT, and its reverse process, MET are two mechanisms influenced by the gut microbiota, which are responsible for intestinal balance and are involved in CRC. On one hand, EMT allows cells to migrate and repair tissue damage, while MET restores the epithelial state, maintaining gut integrity. This balance ensures that the gut can respond to and recover from damage, reducing inflammation and cancer-promoting conditions. It has been reported that certain commensal bacteria produce substances, such as polysaccharide A (PSA), which promote the assembly and reinforce the tight junctions essential for intestinal barrier maintenance. PSA interacts with specific receptors on epithelial cells and increases the expression of proteins related to tight junction formation, thereby fortifying the barrier (Sittipo et al., 2018). In addition, the microbiota competes with potentially harmful microorganisms for space and resources. By occupying niche sites, resident microorganisms prevent the colonization of pathogenic bacteria that can disrupt the epithelial barrier. This colonization resistance preserves the proper functions of the epithelium and reduces the risk of EMT-related pathologies (Ducarmon et al., 2019). In contrast, imbalance or altered composition of the microbiota (known as dysbiosis) can lead to increased intestinal permeability and compromised barrier functions. This can result in the translocation of microbial components, such as lipopolysaccharides (LPS), into the underlying tissues, triggering inflammation and potentially promoting EMT (Wang et al., 2019). IBD is an example of a condition where dysbiosis and a compromised barrier function contribute to EMT-related pathology. IBD causes the infiltration of luminal contents, including bacteria and their products, into the intestinal mucosa. This chronic inflammatory environment can trigger EMT in intestinal epithelial cells and contribute to IBD progression (Yu, 2018) and CRC onset. Overall, the capability of the gut microbiota in maintaining epithelial integrity is critical for preventing the initiation and progression of diseases related to EMT. Moreover, gut microbiota’s capability to regulate the immune response has been linked to EMT and CRC progression. In detail, a higher abundance of *Bacteroides* and *Bacillus faecalis* has been associated with expanding regulatory T cells (Tregs) in CRC microenvironment (Kikuchi et al., 2020), reducing anti-tumor immune responses and allowing the secretion of factors that promote EMT and facilitating cancer metastasis. Moreover, the gut microbiota composition can influence the polarization of tumor-associated macrophages (TAMs) in CRC microenvironment, ranging from anti-tumor (M1-like) to pro-tumor (M2-like) phenotypes. Interestingly, the exogen administration of *E. coli* in mice gut, which mimics microbiota unbalance, leads to LPS secretion and cathepsin K (CTSK) upregulation, which in turn stimulate TLR4 and M2 polarization of TAMs in an mTOR-dependent manner. Highlighting the controversial role of both IL-10 and IL-17 in tumor metastasis (Yu et al., 2024; Zhang et al., 2024); in the CRC context, it has been reported that secretion of CTSK stimulated by the gut microbiota, in turn promotes IL10 and IL17 release from M2 TAMs, promoting CRC invasion and metastasis through NF-κB pathway (Li et al., 2019). The microbiota can also directly interact with epithelial cells through pattern recognition receptors (PRRs) expressed on the cell surface (Li et al., 2022). PRRs, such as Toll-like receptors (TLRs), recognize microbial components and initiate both the innate and adaptive immune responses regulating the balance for host-microorganism symbiosis. Alteration of this equilibrium, such as dysbiosis of the gut microbiota, can alter TRL signaling activating inflammatory and metabolic responses, potentially resulting in diseases such obesity, inflammatory bowel disease (IBD), and colorectal cancer (CRC) (Chen et al., 2024). Among other cellular processes, it has been described that the activation of TLR signaling can trigger downstream pathways affecting EMT. For instance, TLR activation can produce inflammatory mediators that promote EMT or, by contrast, induce anti-inflammatory responses that inhibit EMT (Fang et al., 2022). Additionally, resident gut microorganisms produce a wide array of metabolites, including SCFAs, which are generated by the fermentation of dietary fibers by gut bacteria in the colon and produce acetate, propionate, and butyrate, which impact EMT (Morrison and Preston, 2016). Butyrate is a known Histone deacetylase (HDAC) inhibitor, as it can increase histone acetylation, leading to changes in gene expression. By inhibiting HDACs, butyrate can alter the acetylation status of histones associated with EMT-related genes, thereby modulating their expression and inhibiting EMT. However, metabolite effects are context-dependent and may vary across different cell types and disease conditions (Mrkvicova et al., 2019). Furthermore, the microbiota can produce other metabolites, such as indole derivatives and secondary bile acids, which can impact EMT processes by binding the farnesoid X receptor (FXR) (Zeng et al., 2019). Therefore, metabolites are critical to sustaining the proper microbiota-EMT connection, linking the microbial community to cellular processes involved in tissue homeostasis and disease progression. Of note, the microbiota can also modulate the bioavailability of chemotherapeutic drugs in the gut, with some bacteria able to metabolize molecules, potentially altering their efficacy. It has been reported that some microorganisms can modulate the expression and activity of transmembrane transporters, affecting the intracellular concentration of drugs and their cytotoxic effects. Therefore, EMT may also alter drug metabolism and transport in this context, further altering the chemotherapeutic response (Alexander et al., 2017). The EMT-microbiota interplay in CRC is schematically depicted in Figures 1a–c.

### 5.3 EMT/autophagy inter-relations in CRC

Autophagy and EMT are two physiological mechanisms implicated in cell differentiation and remodeling; therefore, it is not surprising that they cross-react at multiple points and are involved in tumorigenesis. During embryo development, it is well-characterized that EMT-transcription factors (TFs) are continuously expressed; however, in adulthood, their expression is tightly regulated, with autophagy degrading EMT-TFs and contributing to their short half-life (Chen et al., 2019). Accumulating evidence supports an antagonistic role between autophagy and EMT in cancer: on the one hand, EMT promotes mTOR pathway activation with the consequent inhibition of autophagy (Marcucci et al., 2016). Conversely, the BECLIN1 inhibition upregulates SNAIL and SLUG, thus promoting glioblastoma cell migration and invasion (Catalano et al., 2015). Of note, the SQSTM1/p62 accumulation caused by autophagy inhibition stabilizes TWIST1 protein expression, resulting in proliferation and metastasis formation (Han et al., 2022). However, autophagy may favor EMT in other circumstances; for instance, lung cancer cells with co-mutations in KRAS and LKB1 take advantage of the increased acetyl-coA produced during autophagy, supporting SNAIL acetylation and cancer invasion (Han et al., 2022). Despite the intense crosstalk between autophagy and EMT, which has been described in several cancers, only a few reports focus on CRC. For instance, Beclin-1 downregulation has been described to prevent rapamycin-induced autophagy, reducing EMT-related markers’ expression in CRC cell lines (Shen et al., 2018). Accordingly, efficient autophagy flux is required to promote acquiring stem features and chemoresistance linked to EMT. The SOX2-catenin/Beclin1/autophagy axis contributes to the development and chemotherapy resistance in SW480 and SW620 cells (Zhu et al., 2021). However, in another study, AMPK negatively regulates ZEB1 expression and invasion in CRC cell line SW620 while inducing E-cadherin expression (Kan et al., 2016). Kan et al. report that mTOR activation is associated with EMT induction, while AMPK is linked to EMT repression. Thus, the interrelations between EMT and autophagy in tumors appear contextual and cellular-specific.

Finally, it is also essential to consider that autophagy and EMT can indirectly crosstalk through common regulative pathways. For example, it is well-established that hypoxia induces both EMT (Shang et al., 2017) and autophagy (Koustas et al., 2019) in CRC. Indeed, cancer cells sustain hypoxia by upregulating the BCL2 interacting protein 3 (BNIP3)/BNIP3-like (BNIP3L), which in turn induces Beclin-1 dependent autophagy by destabilizing Bcl-2-Beclin-1 complex (Lin et al., 2014). In addition, it has been described that the kinase PRKCA/PKCα, which regulates hypoxia-induced autophagy, also promotes tumor-initiating cells (TICs) renewal, driving CRC initiation and progression (Qureshi-Baig et al., 2020). Similarly, the protein phosphatase PP2A dephosphorylates the prolyl hydroxylase domain-containing protein2 (PHD2) and activates the survival response of CRC cells by stimulating HIF-1α mediated autophagy (Di Conza et al., 2017). Of note, in CRC, hypoxia may regulate EMT through the gene associated with retinoid-interferon-induced mortality-19 (GRIM-19), which blocks EMT by suppressing hypoxia-dependent-autophagy (Zhang et al., 2019), and consistently, it is downregulated in CRC patients (Hao et al., 2015). Finally, in the CRC context, sphingosine kinase 1 (SPHK1) also induces autophagy and promotes CRC invasion and metastasis by modulating the phosphorylation of focal adhesion paxillin (Wu J. N. et al., 2021).

## 6 Gut microbiota-autophagy-EMT crosstalk in CRC

It has been extensively argued how the intestinal microbiota influences both autophagy and EMT in CRC. Figure 1 outlines and integrates several proteins, molecules and pathways, described in the previous paragraphs, and graphically depicts the crosstalk between microbiota-autophagy-EMT involved in CRC onset (panel a), progression (panel b), therapy resistance and cancer recurrence (panel c). Despite the intense crosstalk between autophagy and EMT in several cancers, only a few reports focus on CRC. In this context, BECLIN-1 downregulation has been described to prevent rapamycin-induced autophagy, reducing EMT-related markers’ expression in CRC cell lines (Shen et al., 2018). In addition, BECLIN-1 has been recently described in maintaining intestinal homeostasis in an autophagy-independent manner (Tran et al., 2024). Similarly, the BECLIN-1 interacting protein AMBRA1 (Antonioli et al., 2015) has been linked to intestinal inflammation, with a mechanism independent from autophagy but related to the NF-kB cascade, thus suggesting a possible impact on EMT (Xu et al., 2024). These pieces of evidence suggest a more sophisticated regulation where autophagic proteins can be modified to regulate intestinal EMT, independently from autophagy. In addition, microorganisms can directly promote autophagy and result in the regulation of EMT-related proteins. In this regard, BECLIN-1 expression has been found upregulated in *F. nucleatum* infected CRC cell lines, thus resulting in autophagy activation and E-cadherin downregulation. In line, it has been reported *F. nucleatum* promotes Wnt/B-catenin pathway in CRC (Li et al., 2021), a mechanism extensively described in EMT (Xue et al., 2024). Interestingly, it has been reported that the SOX2-catenin/Beclin1/autophagy axis promotes EMT and chemotherapy resistance of SW480 and SW620 cells (Zhu et al., 2021). Similarly to other cancers, SOX2 promotes EMT by modulating Wnt pathway (Han et al., 2012). Thus, the microbiota could indirectly influence EMT by regulating SOX2, autophagy and Wnt signal.

Interestingly, AMPK, which senses cellular glucose and positively regulates autophagy, has been also described to reduce ZEB1 expression and invasion in CRC cell line SW620 while inducing E-cadherin expression (Kan et al., 2016). In this study, authors reported that the modulation of critical autophagic regulators inversely correlates with EMT. Specifically, AMPK inhibits EMT, while mTOR, a negative regulator of autophagy, is activated to sustain it. In line, it has been described that the gut microbiota extensively regulates AMPK activity by short-chain fatty acids (Sun and Zhu, 2017) (SCFA) and, metformin, which suppresses CRC growth, increases the amount of short-SCFA-producing microbes (Broadfield et al., 2022), as well as induces autophagy by stimulating AMPK-related signaling pathways (Lu et al., 2021). These findings point out how the microbiota composition could influence intestinal glucose abundance, thus impacting AMPK activity, autophagy, and EMT and, in turn, CRC onset and progression.

Finally, it is also essential to consider that autophagy and EMT can indirectly crosstalk through common regulative pathways. For example, it is well-established that hypoxia induces both EMT and autophagy in CRC (Shang et al., 2017). In this regard, intestinal microorganisms are essential to properly maintain the hypoxic environment, which is necessary for nutrient absorption (Singhal and Shah, 2020). In fact, Laís. P. Pral et al. highlight a cycle in which commensal anaerobic bacteria release metabolites that IECs use to generate ATP via mitochondrial respiration, causing oxygen depletion in the environment. Conditions like antibiotics or low-fibre diets that interfere with either microbiota composition or epithelial metabolism can compromise this mutually beneficial interaction, disrupting hypoxia and promoting pathogen colonisation (Pral et al., 2021). Consistent with this, it has been demonstrated that HIF-1β–deficient IECs show an aberrant junctional morphology; on the other hand, the ectopic expression of CLDN1 in HIF-1-deficient cells resolved morphological defects, restoring the barrier function (Di Mattia et al., 2024). Additionally, it is well established that HIFs play a role in cancer progression. Specifically, in human colon cancer tissues, the expression of HIF-1α isoforms—and to a lesser extent, HIF-2α—was associated with the upregulation of VEGF and tumour angiogenesis. However, the loss of HIF-2α expression, but not HIF-1α, was strongly linked to advanced tumour stages, suggesting that HIF isoforms may have different cellular functions in colon cancer. In fact, HIF-1α promoted the growth of SW480 colon cancer cells, while HIF-2α appeared to inhibit growth (Imamura et al., 2009). Therefore, dysbiosis can impact hypoxia-inducible factors (HIFs) in the IECs and, consequently, affect CRC onset and progression. Moreover, it has been reported that cancer cells sustain hypoxia by upregulating the BCL2 interacting protein 3 (BNIP3)/BNIP3-like (BNIP3L), which in turn induces BECLIN-1 dependent autophagy by destabilizing Bcl-2-BECLIN-1 complex (Lin et al., 2014). Also, the kinase PRKCA/PKCα regulates hypoxia-induced autophagy and promotes tumor-initiating cells (TICs) renewal, thus driving CRC initiation and progression (Qureshi-Baig et al., 2020). Early during CRC development, an alteration of the hypoxic state could cause a detrimental alteration of the intestinal microbiota, thus increasing inflammation and promoting cancer development. Similarly, the protein phosphatase PP2A dephosphorylates the prolyl hydroxylase domain-containing protein2 (PHD2) and activates the survival response of CRC cells by stimulating HIF-1α mediated autophagy (Di Conza et al., 2017). Interestingly, hypoxia may also regulate EMT through retinoid-interferon-induced mortality-19 (GRIM-19), which blocks EMT by suppressing hypoxia-dependent-autophagy (Zhang et al., 2019), and consistently, it is downregulated in CRC patients (Hao et al., 2015).

## 7 Microbiota as biomarkers in CRC diagnosis and prognosis

As mentioned, specific alterations in the gut microbiota have been linked to CRC; therefore, detecting these signatures may represent an assessable diagnostic and prognostic approach to facilitate early cancer detection and support patient stratification for specific therapies. However, despite this promising potential, significant challenges persist in translating microbiota-based biomarkers into clinical practice.

**FIGURE 2:**
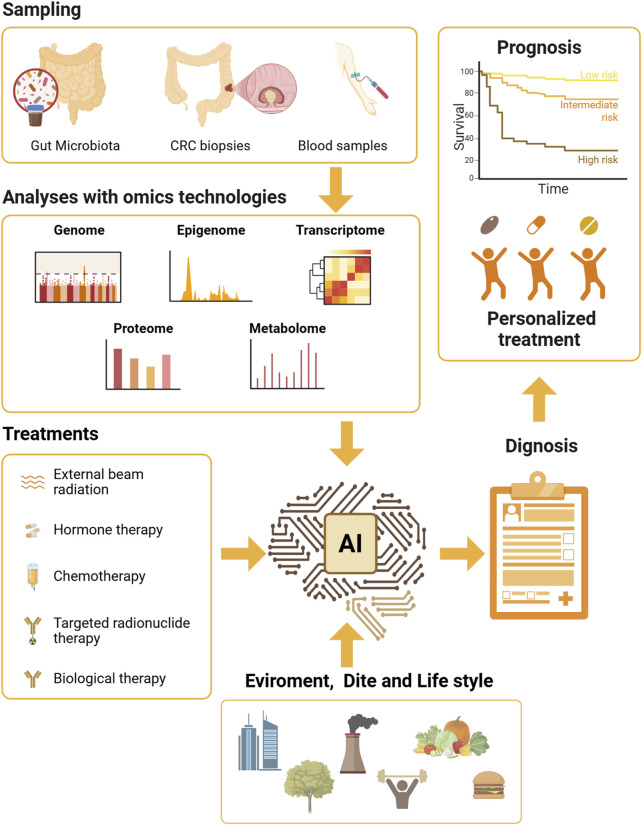
New frontiers in combining Microbiota-derived information for the clinical benefit of CRC patients. This figure outlines a comprehensive framework for precision medicine in CRC, focusing on the integration of gut microbiota analysis with advanced omics technologies. The process begins with data collection from patients, followed by detailed analyses using various omics technologies, including genome, epigenome, transcriptome, proteome, and metabolome. These analyses aim to identify specific biomarkers and molecular profiles that can inform personalised treatment strategies. Personalised treatments have to consider several factors, such as the therapeutic intervention related to CRC treatment, environment, diet, and lifestyle, in shaping the gut microbiota and influencing treatment outcomes. The integration of artificial intelligence (AI) is depicted as a crucial component for enhancing the accuracy of diagnosis and prognosis. AI algorithms analyse the complex data generated from omics technologies to predict patient responses to different treatments and optimise precise therapeutic strategies. This future-oriented approach aims to leverage the synergy between gut microbiota analysis, omics technologies, and AI to develop highly tailored and effective treatment plans, ultimately enhancing the quality of life and survival rates for CRC patients. Created in BioRender. Antonioli, M. (2025) https://BioRender.com/ueon36o.

Similarly to other cancers, early detection of CRC is crucial for improving survival rates; therefore, microbiota-based biomarkers would offer a non-invasive approach to screening. It has been extensively reported that CRC patients possess distinctive gut microbiota signatures compared to healthy controls. As mentioned, *F. nucleatum, B. fragilis,* and colibactin-producing *E. coli* have been consistently associated with CRC development (Zhao et al., 2023). However, several host parameters (e.g., intestinal transit time, inflammation and the body mass index) influence the microbiome composition, reducing the reliability of CRC diagnosis. It has been recently reported (Tito et al., 2024) that these parameters influence gut microbiota composition in a cohort of 589 CRC, reducing the significance of F. *nucleatum* its association with CRC diagnostic groups (healthy, adenoma and carcinoma). By contrast, other microorganisms are not affected by host confounders, thus revealing how the evaluation of several species could represent a valuable diagnostic strategy (*e.g., A. vaginalis*, *D. pneumosintes*, *P. micra*, *P. anaerobius*, *P. asaccharolytica* and *P. intermedia*). This study highlights one of the most crucial aspects of using microbiota in diagnosis, due to its intimate connection with various host parameters. In the next future, the ability to evaluate multiple factors through advanced informatic tools will undoubtedly be pivotal in using microbiota as a biomarker. In this regard, the artificial intelligence, already used to assess several parameters on both CRC patients (Patil et al., 2024) and microbiome (González et al., 2024) separately, could be combined to develop specific models sustaining clinicians for precise diagnosis and treatments (Figure 2). Subsequently, the possibility of characterizing the microbiota composition in the routine CRC screening programs could enhance early detection efforts, especially for high-risk individuals. Nevertheless, validation in large-scale, multi-cohort studies would be required to standardize microbial biomarkers and integrate them with existing diagnostic methods.

Microbiota composition is not only relevant for CRC diagnosis but also for predicting disease progression and patient outcomes, and specific microbial patterns have been associated with tumor stage, metastasis, and treatment response. Notably, it has been recently reported that EMT is actively promoted by *F. nucleatum by* miR-5692a/IL-8, thus facilitating CRC metastatization to liver (Yu et al., 2025). Moreover, *F. nucleatum* presence is linked to reduced overall survival of CRC patients (Kunzmann et al., 2019), while *P. copri* and *F. prausnitzii* exhibit better responses to immune checkpoint inhibitor (ICI) (Chang et al., 2024). More in general, CRC progression is reduced with high human enterotypes (e.g., *Prevotella*) and increased with *Bacteroides* sp., *P. piscolens*, *D. invisus*, and *F. nucleatum* (Huh et al., 2022). Interestingly, seventeen different microorganisms among genera and familia of bacteria have been identified as possible biomarkers for CRC recurrence (Huo et al., 2022), overall highlighting the potential of microbiota analysis for personalized clinical approaches.

In addition, other aspects of using gut microbiota in clinical practice are challenging; for instance, the standardization of sample collection, consisting of their processing and the methods used for the analysis, will be essential to ensure reproducibility and consistency across studies. Furthermore, integrating microbiota analysis into existing CRC diagnostic programs would require cost-effective and scalable methodologies. While next-generation sequencing and metagenomics would offer high-resolution microbial profiling, they are still expensive and time-consuming to be used in the clinical routine. To overcome these limits, the development of rapid and low-cost assays able to detect specific microorganisms (e.g., qPCR-based tests) could increase the chance of using large-scale screening.

Equally significant considerations consist of regulatory and ethical aspects. Indeed, microbiota-based diagnostics provide the management and analysis of human microbiome data and raise concerns about data privacy and patient consent. Formulating guidelines for microbiome research and ensuring ethical standards are imperative for translating clinical practices. Therefore, it would be relevant to refine microbiota-based assays, integrate them with existing diagnostic tools, and explore microbiota-targeted interventions to improve CRC management.

## 8 Therapeutic interventions targeting tumor microbiota in CRC

The evident correlation between microbiota and CRC also opened the possibility of developing therapeutic strategies targeting tumor-associated microorganisms. These approaches involve the use of probiotics, prebiotics, fecal microbiota transplantation (FMT), antibiotics, and the modulation of microbiota to enhance the efficacy of immunotherapy.

Probiotics and prebiotics have gained attention for their potential to modulate gut microbiota and improve CRC treatment outcomes (Moreira et al., 2024). Probiotics are live microorganisms that confer health benefits by restoring microbial balance, while prebiotics are dietary-derived fibers promoting the growth of beneficial bacteria. As mentioned, several probiotic strains, including *Lactobacillus* and *Bifidobacterium*, exhibit anti-cancer properties by enhancing intestinal barrier integrity, reducing inflammation, and modulating immune responses and are objects of several clinical trials for the prevention and treatment of CRC. Recently, the use of naïve microorganisms has been partially overcome by engineered bacteria, which have been modified significantly for their capability of converting prodrugs into cytotoxic products at the tumor site, specifically targeting the tumor microenvironment (TME) and reducing adverse effects on organisms (Han et al., 2024). Among accepted prebiotics (Gibson et al., 2017), insulin and fructooligosaccharides (FOS) have been described as stimulating the growth of beneficial bacteria (Moreira et al., 2024), and leading to the production of short-chain fatty acids (SCFAs) with anti-inflammatory and anti-tumor effects in CRC (Donohoe et al., 2014). Therefore, both pro- and prebiotics have a relevant clinical potential in actively sustaining current CRC therapies.

Similarly, FMT is an emerging therapeutic approach that involves transferring microbiota from a healthy donor into a CRC patient to restore microbial balance and sustain the therapy (Su et al., 2024). Initially developed for treating recurrent *C. difficile* infections, FMT is now being investigated for its potential in cancer therapy. Studies have shown that FMT can modulate the tumor microenvironment and improve immune responses in murine models (Yu et al., 2023). In preclinical models, FMT from healthy donors has been associated with reduced tumor growth and enhanced efficacy of immune checkpoint inhibitors (ICIs) (Kang and Cai, 2021). Despite being very promising, several challenges remain in standardizing FMT procedures, which concern safeguarding donor safety, and the long-term effects. Current clinical trials are underway to assess FMT’s effectiveness in treating CRC and its potential use as an adjunct to immunotherapy.

Antibiotics have also been explored to reshape tumor-associated microbiota and improve cancer treatment outcomes. Whether some antibiotics can selectively affect microorganisms directly involved in CRC (*e.g., F. nucleatum*), their indiscriminate use may also target beneficial microbial communities, leading to dysbiosis and increased inflammation, thus facilitating CRC onset. Studies have reported that long-term antibiotic exposure may elevate CRC onset (Perrott et al., 2021) and recurrence (Hilmi et al., 2025), therefore the development of precision-targeted antibiotics or antimicrobial peptides (Jia et al., 2023) would be necessary to target tumor-promoting bacteria, while specifically preserving beneficial microbiota.

**FIGURE 3:**
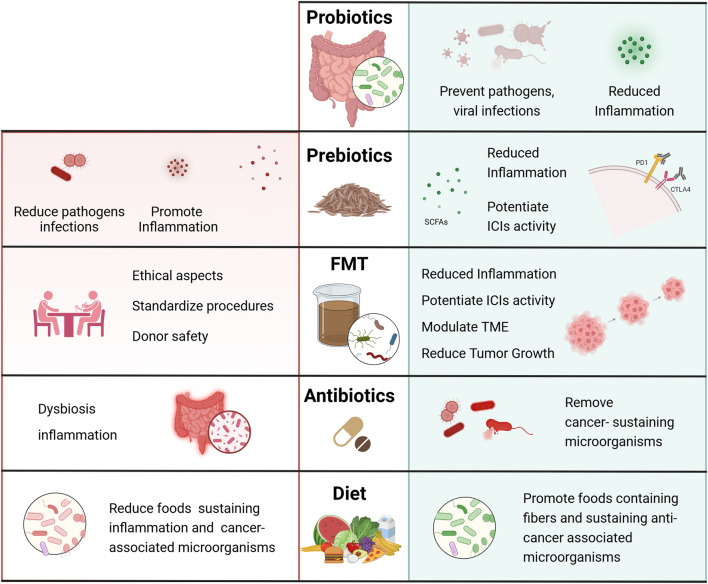
Benefits and drawbacks of therapeutic interventions targeting the microbiota for CRC treatment. Schematic representation of possible interventions that target the microbiota and could support canonical therapy in colorectal cancer patients. The administration of probiotics and prebiotics has shown potential in both preventing and inhibiting CRC development by modulating the gut microbiota composition, mitigating inflammation, and influencing the host’s immune response. FMT, which involves transferring fecal material from a healthy donor to a patient recipient, is implemented to restore microbiome balance in patients, in order to reduce inflammation and tumor growth. Although FMT is emerging as a promising therapeutic tool in CRC treatment, FMT standardization procedures, involving rigorous donor screening, stool processing, and administration, need to be further developed to improve safety and efficacy of this treatment. Administration of antibiotics should be carefully evaluated as, even though they can target harmful bacteria associated with CRC, they can also disrupt the overall microbiome balance, potentially worsening inflammation and dysbiosis. Finally, a diet rich in foods containing fibers may be regarded as an auxiliary treatment strategy to prevent CRC risk or improve outcomes of CRC patients. Created in BioRender. Antonioli, M. (2025) https://BioRender.com/ja3od5h.

All these approaches hold the potential for enhancing CRC treatment efficacy without inducing harmful microbial imbalances and harnessing the increasing knowledge of the interaction between the gut microbiota, host responding mechanisms and CRC pathology (Figure 3).

## 9 Conclusion and prospects

Beyond genetic mutations and oncogenic viruses (Vescovo et al., 2020), recent findings suggest that microbiota contributes to CRC development through effects on inflammation, immunity, DNA damage, autophagy, and EMT. Advanced sequencing technologies have identified microbial signatures in CRC, notably *F. nucleatum*, *B. fragilis*, and colibactin-producing *E. coli*, along with metabolites like SCFAs and bile acids, which modulate tumor behaviour (Zhang et al., 2025). Recent discoveries highlight how EMT predominantly plays a role in the progression and metastasis of CRC, whereas autophagy is mainly involved in cancer onset, chemoresistance, and recurrence. In this context, microbiota modulates both pathways and immunity response, favoring or constraining CRC pathology depending on its composition and balance. To date, AMPK, NF-κB, mTOR, and hypoxia are emerging as fundamental mechanisms in maintaining the proper intestinal balance and, by regulating both autophagy and EMT, could represent the main link in this crosstalk. However, a more significant effort would be helpful in understanding which microbiota microorganisms support intestinal health, limiting dysbiosis and inflammation and promoting a proper equilibrium between autophagy and EMT. Indeed, while intestinal dysbiosis increases EMT and inflammation and modulates autophagy, activating cancer-related pathways, the appropriate maintenance of the microbiota balance and their metabolites can constrain cancer progression. Overall, understanding the complex interplay between the microbiota, EMT regulation, autophagy, and cancer progression could open new perspectives also in CRC prevention and treatment. In this direction, Metformin has been shown to reduce CRC risk (Higurashi and Nakajima, 2018; Lu et al., 2021), potentially through its modulation of autophagy, microbiota composition, and EMT, thus supporting the relevance of their interplay in CRC pathophysiology. Understanding the molecular mediators of this crosstalk could reveal novel therapeutic targets.

In addition, microbial profiling through NGS and metagenomic analysis has enabled the identification of microbial signatures distinguishing CRC patients from healthy individuals. Fecal microbiota-based tests have shown the potential to complement traditional CRC screening methods (e.g., colonoscopy and fecal occult blood test) since the microbiota composition correlates with tumor aggressiveness, metastasis, and treatment response, thus highlighting its potential in patient prognosis. However, clinical translation is limited by inter-individual variability, lack of standardization, and the difficulty in distinguishing causative microbial shifts from incidental ones. Beyond its role in diagnosis and prognosis, microbiota is emerging as a promising therapeutic target in CRC. To date, specific probiotics and prebiotics have been shown to ensure the microbial balance, therefore several methods have been explored to modify microbiota composition and improve treatment results, thus enhancing anti-tumor immunity. To this regards, beneficial bacteria such as *Bifidobacterium* and *Lactobacillus* are beneficial in limiting CRC progression by modulating immune responses and reducing inflammation, while prebiotics such as inulin and fructooligosaccharides (FOS) promote the growth of beneficial microorganisms and the production of tumor-suppressive metabolites (Han et al., 2024). Fecal microbiota transplantation (FMT) is also being investigated to restore a healthy microbiota composition in CRC patients (Yu et al., 2023). Antibiotics have also been explored to eliminate tumour-promoting bacteria (e.g., *F. Nucleatum*). However, their use is a double-edged sword; indeed, the indiscriminate use of antibiotics promotes dysbiosis and inflammation. Therefore, to sustain cancer therapy, it would be necessary to develop specific molecules which target detrimental microorganisms while preserving beneficial ones. Microbiota modulation may enhance immunotherapy effectiveness in CRC (Zhao et al., 2023). Key challenges include mechanistic understanding and ethical concerns, particularly regarding FMT safety and regulation. In light of reported considerations, future research should focus on developing precision microbiome-based medicine tailored to individual patient microbiota profiles. In line, the integration of data from microbiota screening with other omics technologies (e.g., metabolomics and transcriptomics) could give new insights into the microbiota’s role in cancer, as well as inform novel therapeutic targets. Despite significant challenges remain, ongoing research and technological advancements are paving the way for microbiome-based precision medicine in CRC. By harnessing the power of the microbiome, future cancer therapies may become more effective, personalized, and integrative, ultimately leading to better patient care and improved survival rates.

## References

[B1] Alexander J. L. Wilson I. D. Teare J. Marchesi J. R. Nicholson J. K. Kinross J. M. (2017). Gut microbiota modulation of chemotherapy efficacy and toxicity. Nat. Rev. Gastroenterol. Hepatol. 14, 356–365. 10.1038/nrgastro.2017.20 28270698

[B2] Antonioli M. Albiero F. Fimia G. M. Piacentini M. (2015). AMBRA1-regulated autophagy in vertebrate development. Int. J. Dev. Biol. 59, 109–117. 10.1387/ijdb.150057mp 26374532

[B3] Arnone A. A. Cook K. L. (2022). Gut and breast microbiota as endocrine regulators of hormone receptor-positive breast cancer risk and therapy response. Endocrinology 164, bqac177. 10.1210/ENDOCR/BQAC177 36282876 PMC9923803

[B4] Azevedo M. M. Pina-Vaz C. Baltazar F. (2020). Microbes and cancer: friends or faux? Int. J. Mol. Sci. 21, 3115. 10.3390/IJMS21093115 32354115 PMC7247677

[B5] Beilankouhi E. A. V. Sajadi M. A. Alipourfard I. Hassani P. Valilo M. Safaralizadeh R. (2023). Role of the ER-induced UPR pathway, apoptosis, and autophagy in colorectal cancer. Pathol. Res. Pract. 248, 154706. 10.1016/J.PRP.2023.154706 37499516

[B6] Benítez-Páez A. Gómez del Pulgar E. M. Sanz Y. (2017). The glycolytic versatility of Bacteroides uniformis CECT 7771 and its genome response to oligo and polysaccharides. Front. Cell Infect. Microbiol. 7, 291727. 10.3389/FCIMB.2017.00383/BIBTEX PMC5609589 28971068

[B7] Bian J. Dannappel M. Wan C. Firestein R. (2020). Transcriptional regulation of wnt/β-catenin pathway in colorectal cancer. Cells 9, 2125. 10.3390/cells9092125 32961708 PMC7564852

[B8] Broadfield L. A. Saigal A. Szamosi J. C. Hammill J. A. Bezverbnaya K. Wang D. (2022). Metformin-induced reductions in tumor growth involves modulation of the gut microbiome. Mol. Metab. 61, 101498. 10.1016/J.MOLMET.2022.101498 35452877 PMC9096669

[B9] Bullman S. Pedamallu C. S. Sicinska E. Clancy T. E. Zhang X. Cai D. (2017). Analysis of fusobacterium persistence and antibiotic response in colorectal cancer. Science 1979, 1443–1448. 10.1126/science.aal5240 PMC5823247 29170280

[B10] Cai L. Zhu H. Mou Q. Wong P. Y. Lan L. Ng C. W. K. (2024). Integrative analysis reveals associations between oral microbiota dysbiosis and host genetic and epigenetic aberrations in oral cavity squamous cell carcinoma. npj Biofilms Microbiomes 10 (1), 39–16. 10.1038/s41522-024-00511-x 38589501 PMC11001959

[B11] Castaneda C. Castillo M. Sanchez J. Casavilca S. Sanchez J. A Bernabe L. (2020). Detection of Helicobacter pylori in gastric cancer tissue through histopathology, immunohistochemistry and real-time reverse transcription-PCR. Future Microbiol. 15, 1131–1137. 10.2217/FMB-2019-0280 32954850

[B12] Catalano M. D’Alessandro G. Lepore F. Corazzari M. Caldarola S. Valacca C. (2015). Autophagy induction impairs migration and invasion by reversing EMT in glioblastoma cells. Mol. Oncol. 9, 1612–1625. 10.1016/J.MOLONC.2015.04.016 26022108 PMC5528793

[B13] Cavallucci V. Palucci I. Fidaleo M. Mercuri A. Masi L. Emoli V. (2022). Proinflammatory and cancer-promoting pathobiont Fusobacterium nucleatum directly targets colorectal cancer stem cells. Biomolecules 12, 1256. 10.3390/biom12091256 36139097 PMC9496236

[B14] Centelles J. J. (2012). General aspects of colorectal cancer. ISRN Oncol. 2012, 139268. 10.5402/2012/139268 23209942 PMC3504424

[B15] Chang J. W. C. Hsieh J. J. Tsai C. Y. Chiu H. Y. Lin Y. F. Wu C. E. (2024). Gut microbiota and clinical response to immune checkpoint inhibitor therapy in patients with advanced cancer. Biomed. J. 47, 100698. 10.1016/J.BJ.2024.100698 38280521 PMC11399570

[B16] Chen J. Pitmon E. Wang K. (2017). Microbiome, inflammation and colorectal cancer. Semin. Immunol. 32, 43–53. 10.1016/j.smim.2017.09.006 28982615

[B17] Chen H. T. Liu H. Mao M. J. Tan Y. Mo X. Q. Meng X. J. (2019). Crosstalk between autophagy and epithelial-mesenchymal transition and its application in cancer therapy. Mol. Cancer 18, 101. 10.1186/s12943-019-1030-2 31126310 PMC6533683

[B18] Chen Y. Chen Y. Zhang J. Cao P. Su W. Deng Y. (2020). Fusobacterium nucleatum promotes metastasis in colorectal cancer by activating autophagy signaling *via* the upregulation of CARD3 expression. Theranostics 10, 323–339. 10.7150/thno.38870 31903123 PMC6929621

[B19] Chen M. Lin W. Li N. Wang Q. Zhu S. Zeng A. (2022). Therapeutic approaches to colorectal cancer *via* strategies based on modulation of gut microbiota. Front. Microbiol. 13, 945533. 10.3389/fmicb.2022.945533 35992678 PMC9389535

[B20] Chen Z. Guan D. Wang Z. Li X. Dong S. Huang J. (2023). Microbiota in cancer: molecular mechanisms and therapeutic interventions. MedComm (Beijing) 4, e417. 10.1002/MCO2.417 PMC10626288 37937304

[B21] Chen L. Zhang L. Hua H. Liu L. Mao Y. Wang R. (2024). Interactions between toll‐like receptors signaling pathway and gut microbiota in host homeostasis. Immun. Inflamm. Dis. 12, e1356. 10.1002/IID3.1356 39073297 PMC11284964

[B22] Cheng Y. Ling Z. Li L. (2020). The intestinal microbiota and colorectal cancer. Front. Immunol. 11, 615056. 10.3389/FIMMU.2020.615056 33329610 PMC7734048

[B23] Cho D. H. Jo Y. K. Kim S. C. Park I. J. Kim J. C. (2012). Down-regulated expression of ATG5 in colorectal cancer. Anticancer Res. 32, 4091–4096. 22993366

[B24] Coppola D. Khalil F. Eschrich S. A. Boulware D. Yeatman T. Wang H. G. (2008). Down-regulation of bax-interacting factor-1 in colorectal adenocarcinoma. Cancer 113, 2665–2670. 10.1002/cncr.23892 18833585 PMC2614910

[B25] Costea P. I. Hildebrand F. Manimozhiyan A. Bäckhed F. Blaser M. J. Bushman F. D. (2017). Enterotypes in the landscape of gut microbial community composition. Nat. Microbiol. 3, 8–16. 10.1038/s41564-017-0072-8 29255284 PMC5832044

[B26] Dadgar-Zankbar L. Elahi Z. Shariati A. Khaledi A. Razavi S. Khoshbayan A. (2024). Exploring the role of Fusobacterium nucleatum in colorectal cancer: implications for tumor proliferation and chemoresistance. Cell Commun. Signal. 22 (1), 547–16. 10.1186/S12964-024-01909-Y 39548531 PMC11566256

[B27] Dalmasso G. Cougnoux A. Faïs T. Bonnin V. Mottet-Auselo B. Nguyen H. T. T. (2024). Colibactin-producing *Escherichia coli* enhance resistance to chemotherapeutic drugs by promoting epithelial to mesenchymal transition and cancer stem cell emergence. Gut Microbes 16, 2310215. 10.1080/19490976.2024.2310215 38374654 PMC10880512

[B28] Devenport S. N. Singhal R. Radyk M. D. Taranto J. G. Kerk S. A. Chen B. (2021). Colorectal cancer cells utilize autophagy to maintain mitochondrial metabolism for cell proliferation under nutrient stress. JCI Insight 6, e138835. 10.1172/jci.insight.138835 34138755 PMC8328084

[B29] Di Conza G. Trusso Cafarello S. Loroch S. Mennerich D. Deschoemaeker S. Di Matteo M. (2017). The mTOR and PP2A pathways regulate PHD2 phosphorylation to fine-tune HIF1α levels and colorectal cancer cell survival under hypoxia. Cell Rep. 18, 1699–1712. 10.1016/J.CELREP.2017.01.051 28199842 PMC5318657

[B30] Di Mattia M. Sallese M. Neri M. Lopetuso L. R. (2024). Hypoxic functional regulation pathways in the GI tract: focus on the HIF-1α and microbiota’s crosstalk. Inflamm. Bowel Dis. 30, 1406–1418. 10.1093/IBD/IZAE046 38484200

[B31] Di Mattia M. Sallese M. Lopetuso L. R. (2025). The interplay between gut microbiota and the unfolded protein response: implications for intestinal homeostasis preservation and dysbiosis-related diseases. Microb. Pathog. 200, 107279. 10.1016/j.micpath.2025.107279 39761770

[B32] Donohoe D. R. Holley D. Collins L. B. Montgomery S. A. Whitmore A. C. Hillhouse A. (2014). A gnotobiotic mouse model demonstrates that dietary fiber protects against colorectal tumorigenesis in a microbiota- and butyrate-dependent manner. Cancer Discov. 4, 1387–1397. 10.1158/2159-8290.CD-14-0501 25266735 PMC4258155

[B33] Ducarmon Q. R. Zwittink R. D. Hornung B. V. H. van Schaik W. Young V. B. Kuijper E. J. (2019). Gut microbiota and colonization resistance against bacterial enteric infection. Microbiol. Mol. Biol. Rev. 83, e00007-19. 10.1128/MMBR.00007-19 31167904 PMC6710460

[B34] Fang Y. Yan C. Zhao Q. Zhao B. Liao Y. Chen Y. (2022). The association between gut microbiota, toll-like receptors, and colorectal cancer. Clin. Med. Insights Oncol. 16, 11795549221130549. 10.1177/11795549221130549 36338264 PMC9634190

[B35] Fitzwalter B. E. Towers C. G. Sullivan K. D. Andrysik Z. Hoh M. Ludwig M. (2018). Autophagy inhibition mediates apoptosis sensitization in cancer therapy by relieving FOXO3a turnover. Dev. Cell 44, 555–565. 10.1016/J.DEVCEL.2018.02.014 29533771 PMC5866042

[B36] Foerster E. G. Mukherjee T. Cabral-Fernandes L. Rocha J. D. B. Girardin S. E. Philpott D. J. (2022). How autophagy controls the intestinal epithelial barrier. Autophagy 18, 86–103. 10.1080/15548627.2021.1909406 33906557 PMC8865220

[B37] Fritz T. Niederreiter L. Adolph T. Blumberg R. S. Kaser A. (2011). Crohn’s disease: NOD2, autophagy and ER stress converge. Gut 60, 1580–1588. 10.1136/GUT.2009.206466 21252204 PMC3897479

[B38] Fukushima S. Shimohata T. Inoue Y. Kido J. Uebanso T. Mawatari K. (2022). Recruitment of LC3 by Campylobacter jejuni to bacterial invasion site on host cells *via* the Rac1-Mediated signaling pathway. Front. Cell Infect. Microbiol. 12, 829682. 10.3389/fcimb.2022.829682 35310852 PMC8927770

[B39] Gibson G. R. Hutkins R. Sanders M. E. Prescott S. L. Reimer R. A. Salminen S. J. (2017). Expert consensus document: the international scientific association for probiotics and prebiotics (ISAPP) consensus statement on the definition and scope of prebiotics. Nat. Rev. Gastroenterology Hepatology 14 (8), 491–502. 10.1038/nrgastro.2017.75 28611480

[B40] González A. Fullaondo A. Odriozola I. Odriozola A. (2024). Microbiota and beneficial metabolites in colorectal cancer. cancer 112, 367–409. 10.1016/BS.ADGEN.2024.08.002 39396841

[B41] Goodman B. Gardner H. (2018). The microbiome and cancer. J. Pathology 244, 667–676. 10.1002/path.5047 29377130

[B42] Gou H. Zeng R. Lau H. C. H. Yu J. (2024). Gut microbial metabolites: shaping future diagnosis and treatment against gastrointestinal cancer. Pharmacol. Res. 208, 107373. 10.1016/J.PHRS.2024.107373 39197712

[B43] Grimm W. A. Messer J. S. Murphy S. F. Nero T. Lodolce J. P. Weber C. R. (2016). The Thr300Ala variant in ATG16L1 is associated with improved survival in human colorectal cancer and enhanced production of type I interferon. Gut 65, 456–464. 10.1136/gutjnl-2014-308735 25645662 PMC4789828

[B44] Guinney J. Dienstmann R. Wang X. De Reyniès A. Schlicker A. Soneson C. (2015). The consensus molecular subtypes of colorectal cancer. Nat. Med. 21, 1350–1356. 10.1038/nm.3967 26457759 PMC4636487

[B45] Guo J. Y. Chen H. Y. Mathew R. Fan J. Strohecker A. M. Karsli-Uzunbas G. (2011). Activated ras requires autophagy to maintain oxidative metabolism and tumorigenesis. Genes Dev. 25, 460–470. 10.1101/GAD.2016311 21317241 PMC3049287

[B46] Guo K. Wang P. Zhang L. Zhou Y. Dai X. Yan Y. (2021). Transcription factor POU4F2 promotes colorectal cancer cell migration and invasion through hedgehog-mediated epithelial-mesenchymal transition. Cancer Sci. 112, 4176–4186. 10.1111/cas.15089 34327778 PMC8486210

[B47] Han X. Fang X. Lou X. Hua D. Ding W. Foltz G. (2012). Silencing SOX2 induced mesenchymal-epithelial transition and its expression predicts liver and lymph node metastasis of CRC patients. PLoS One 7, e41335. 10.1371/JOURNAL.PONE.0041335 22912670 PMC3422347

[B48] Han Y. Xue X. F. Shen H. G. Guo X. B. Wang X. Yuan B. (2014). Prognostic significance of beclin-1 expression in colorectal cancer: a meta-analysis. Asian Pac. J. Cancer Prev. 15, 4583–4587. 10.7314/APJCP.2014.15.11.4583 24969889

[B49] Han J. H. Kim Y. K. Kim H. Lee J. Oh M. J. Kim S. B. (2022). Snail acetylation by autophagy-derived acetyl-coenzyme A promotes invasion and metastasis of KRAS-LKB1 co-mutated lung cancer cells. Cancer Commun. (Lond) 42, 716–749. 10.1002/CAC2.12332 35838183 PMC9395322

[B50] Han H. Zhang Y. Tang H. Zhou T. Khan A. A. Han H. (2024). A review of the use of native and engineered probiotics for colorectal cancer therapy. Int. J. Mol. Sci. 25, 3896. 10.3390/IJMS25073896 38612706 PMC11011422

[B51] Hao M. Shu Z. Sun H. Sun R. Wang Y. Liu T. (2015). GRIM-19 expression is a potent prognostic marker in colorectal cancer. Hum. Pathol. 46, 1815–1820. 10.1016/j.humpath.2015.07.020 26363526

[B52] Haruki K. Kosumi K. Hamada T. Twombly T. S. Väyrynen J. P. Kim S. A. (2020). Association of autophagy status with amount of Fusobacterium nucleatum in colorectal cancer. J. Pathology 250, 397–408. 10.1002/path.5381 PMC7282529 31880318

[B53] He Z. Gharaibeh R. Z. Newsome R. C. Pope J. L. Dougherty M. W. Tomkovich S. (2019). Campylobacter jejuni promotes colorectal tumorigenesis through the action of cytolethal distending toxin. Gut 68, 289–300. 10.1136/gutjnl-2018-317200 30377189 PMC6352414

[B54] He F. Zheng Y. Elsabagh M. Fan K. Zha X. Zhang B. (2025). Gut microbiota modulate intestinal inflammation by endoplasmic reticulum stress-autophagy-cell death signaling axis. J. Anim. Sci. Biotechnol. 16, 63. 10.1186/S40104-025-01196-8 40312439 PMC12046778

[B55] Higurashi T. Nakajima A. (2018). Metformin and colorectal cancer. Front. Endocrinol. (Lausanne) 9, 622. 10.3389/FENDO.2018.00622 30405532 PMC6205961

[B56] Hilmi M. Khati I. Turpin A. Andremont A. Burdet C. Grall N. (2025). Association between the antibiotics use and recurrence in patients with resected colorectal cancer: EVADER-1, a nation-wide pharmaco-epidemiologic study. Dig. Liver Dis. 57, 89–96. 10.1016/J.DLD.2024.07.030 39232868

[B57] Hou Y. Li J. Ying S. (2023). Tryptophan metabolism and gut microbiota: a novel regulatory axis integrating the microbiome, immunity, and cancer. Metabolites 13, 1166. 10.3390/METABO13111166 37999261 PMC10673612

[B58] Hu F. Song D. Yan Y. Huang C. Shen C. Lan J. (2021). IL-6 regulates autophagy and chemotherapy resistance by promoting BECN1 phosphorylation. Nat. Commun. 12 (1), 3651–14. 10.1038/s41467-021-23923-1 34131122 PMC8206314

[B59] Huang J. Liu W. Kang W. He Y. Yang R. Mou X. (2022). Effects of microbiota on anticancer drugs: current knowledge and potential applications. EBioMedicine 83, 104197–32000096. 10.1016/j.ebiom.2022.104197 35933808 PMC9358415

[B60] Huh J. W. Kim M. J. Kim J. Lee H. G. Ryoo S. B. Ku J. L. (2022). Enterotypical prevotella and three novel bacterial biomarkers in preoperative stool predict the clinical outcome of colorectal cancer. Microbiome 10, 203. 10.1186/S40168-022-01388-8 36443754 PMC9703702

[B61] Huo R. X. Wang Y. J. Hou S. B. Wang W. Zhang C. Z. Wan X. H. (2022). Gut mucosal microbiota profiles linked to colorectal cancer recurrence. World J. Gastroenterol. 28, 1946–1964. 10.3748/WJG.V28.I18.1946 35664963 PMC9150055

[B62] Imamura T. Kikuchi H. Herraiz M. T. Park D. Y. Mizukami Y. Mino-Kenduson M. (2009). HIF-1alpha and HIF-2alpha have divergent roles in Colon cancer. Int. J. Cancer 124, 763–771. 10.1002/IJC.24032 19030186 PMC2682346

[B63] Jans M. Vereecke L. (2024). A guide to germ-free and gnotobiotic mouse technology to study health and disease. FEBS J. 292, 1228–1251. 10.1111/FEBS.17124 38523409

[B64] Jia F. Yu Q. Wang R. Zhao L. Yuan F. Guo H. (2023). Optimized antimicrobial peptide Jelleine-I derivative Br-J-I inhibits Fusobacterium nucleatum to suppress colorectal cancer progression. Int. J. Mol. Sci. 24, 1469. 10.3390/IJMS24021469 36674985 PMC9865857

[B65] Jiang H. Zhang Q. (2024). Gut microbiota influences the efficiency of immune checkpoint inhibitors by modulating the immune system (review). Oncol. Lett. 27, 87. 10.3892/OL.2024.14221 38249807 PMC10797324

[B66] Jiang H. Li L. Bao Y. Cao X. Ma L. (2024). Microbiota in tumors: new factor influencing cancer development. Cancer Gene Ther. 31 (12), 1773–1785. 10.1038/s41417-024-00833-0 39342031

[B67] Jo Y. K. Kim S. C. Park I. J. Park S. J. Jin D. H. Hong S. W. (2012). Increased expression of ATG10 in colorectal cancer is associated with lymphovascular invasion and lymph node metastasis. PLoS One 7, e52705. 10.1371/journal.pone.0052705 23285162 PMC3527592

[B68] Jochum L. Stecher B. (2020). Label or concept – what is a pathobiont? Trends Microbiol. 28, 789–792. 10.1016/j.tim.2020.04.011 32376073

[B69] Johansen T. Lamark T. (2011). Selective autophagy mediated by autophagic adapter proteins. Autophagy 7, 279–296. 10.4161/AUTO.7.3.14487 21189453 PMC3060413

[B70] Jurjus A. Eid A. Al Kattar S. Zeenny M. N. Gerges-Geagea A. Haydar H. (2016). Inflammatory bowel disease, colorectal cancer and type 2 diabetes mellitus: the links. BBA Clin. 5, 16–24. 10.1016/j.bbacli.2015.11.002 27051585 PMC4802401

[B71] Jyoti Dey P. (2025). Mechanisms and implications of the gut microbial modulation of intestinal metabolic processes. npj Metabolic Health Dis. 3 (1), 24–19. 10.1038/s44324-025-00066-1 PMC12441142 40604123

[B72] Kadosh E. Snir-Alkalay I. Venkatachalam A. May S. Lasry A. Elyada E. (2020). The gut microbiome switches mutant p53 from tumour-suppressive to oncogenic. Nature 586, 133–138. 10.1038/s41586-020-2541-0 32728212 PMC7116712

[B73] Kan J. Y. Yen M. C. Wang J. Y. Wu D. C. Chiu Y. J. Ho Y. W. (2016). Nesfatin-1/Nucleobindin-2 enhances cell migration, invasion, and epithelial-mesenchymal transition *via* LKB1/AMPK/TORC1/ZEB1 pathways in colon cancer. Oncotarget 7, 31336–31349. 10.18632/ONCOTARGET.9140 27150059 PMC5058760

[B74] Kang Y. B. Cai Y. (2021). Faecal microbiota transplantation enhances efficacy of immune checkpoint inhibitors therapy against cancer. World J. Gastroenterol. 27, 5362–5375. 10.3748/WJG.V27.I32.5362 34539138 PMC8409158

[B75] Kapoor S. Padwad Y. S. (2023). Phloretin suppresses intestinal inflammation and maintained epithelial tight junction integrity by modulating cytokines secretion in *in vitro* model of gut inflammation. Cell Immunol. 391-392, 104754–392. 10.1016/j.cellimm.2023.104754 37506521

[B76] Kikuchi T. Mimura K. Ashizawa M. Okayama H. Endo E. Saito K. (2020). Characterization of tumor-infiltrating immune cells in relation to microbiota in colorectal cancers. Cancer Immunol. Immunother. 69, 23–32. 10.1007/s00262-019-02433-6 31768581 PMC11027812

[B77] Klaassen C. D. Cui J. Y. (2015). Review: mechanisms of how the intestinal microbiota alters the effects of drugs and bile acids. Drug Metabolism Dispos. 43, 1505–1521. 10.1124/dmd.115.065698 PMC4576672 26261286

[B78] Klionsky D. J. Abdelmohsen K. Abe A. Abedin M. J. Abeliovich H. Arozena A. A. (2016). Guidelines for the use and interpretation of assays for monitoring autophagy (3rd edition). Autophagy 12, 1–222. 10.1080/15548627.2015.1100356 26799652 PMC4835977

[B79] Klionsky D. J. Petroni G. Amaravadi R. K. Baehrecke E. H. Ballabio A. Boya P. (2021). Autophagy in major human diseases. EMBO J. 40, e108863. 10.15252/embj.2021108863 34459017 PMC8488577

[B80] Koustas E. Sarantis P. Kyriakopoulou G. Papavassiliou A. G. Karamouzis M. V. (2019). The interplay of autophagy and tumor microenvironment in colorectal cancer—ways of enhancing immunotherapy action. Cancers (Basel) 11, 533. 10.3390/cancers11040533 31013961 PMC6520891

[B81] Kunzmann A. T. Proença M. A. Jordao H. W. Jiraskova K. Schneiderova M. Levy M. (2019). Fusobacterium nucleatum tumor DNA levels are associated with survival in colorectal cancer patients. Eur. J. Clin. Microbiol. and Infect. Dis. 38, 1891–1899. 10.1007/S10096-019-03649-1 31367996 PMC6778531

[B82] Larabi A. Barnich N. Nguyen H. T. T. (2020). New insights into the interplay between autophagy, gut microbiota and inflammatory responses in IBD. Autophagy 16, 38–51. 10.1080/15548627.2019.1635384 31286804 PMC6984609

[B83] Lévy J. Cacheux W. Bara M. A. L’Hermitte A. Lepage P. Fraudeau M. (2015). Intestinal inhibition of Atg7 prevents tumour initiation through a microbiome-influenced immune response and suppresses tumour growth. Nat. Cell Biol. 17, 1062–1073. 10.1038/ncb3206 26214133

[B84] Li B. X. Li C. Y. Peng R. Q. Wu X. J. Wang H. Y. Wan D. S. (2009). The expression of beclin 1 is associated with favorable prognosis in stage IIIB colon cancers. Autophagy 5, 303–306. 10.4161/auto.5.3.7491 19066461

[B85] Li R. Zhou R. Wang H. Li W. Pan M. Yao X. (2019). Gut microbiota-stimulated cathepsin K secretion mediates TLR4-dependent M2 macrophage polarization and promotes tumor metastasis in colorectal cancer. Cell Death Differ. 26, 2447–2463. 10.1038/s41418-019-0312-y 30850734 PMC6889446

[B86] Li X. Huang J. Yu T. Fang X. Lou L. Xin S. (2021). Fusobacterium nucleatum promotes the progression of colorectal cancer through Cdk5-Activated Wnt/β-Catenin signaling. Front. Microbiol. 11, 545251. 10.3389/fmicb.2020.545251 33488528 PMC7815597

[B87] Li M. Zhang R. Li J. Li J. (2022). The role of C-Type lectin receptor signaling in the intestinal microbiota-inflammation-cancer axis. Front. Immunol. 13, 894445. 10.3389/FIMMU.2022.894445 35619716 PMC9127077

[B88] Li Q. Geng S. Luo H. Wang W. Mo Y.-Q. Luo Q. (2024). Signaling pathways involved in colorectal cancer: pathogenesis and targeted therapy. Signal Transduct. Target. Ther. 9 (1), 266–48. 10.1038/s41392-024-01953-7 39370455 PMC11456611

[B89] Liang C. Feng P. Ku B. Dotan I. Canaani D. Oh B. H. (2006). Autophagic and tumour suppressor activity of a novel Beclin1-binding protein UVRAG. Nat. Cell Biol. 8, 688–699. 10.1038/ncb1426 16799551

[B90] Liao H. Zhang L. Lu S. Li W. Dong W. (2022). KIFC3 promotes proliferation, migration, and invasion in colorectal cancer *via* PI3K/AKT/mTOR signaling pathway. Front. Genet. 13, 848926. 10.3389/fgene.2022.848926 35812733 PMC9257096

[B91] Lin A. Yao J. Zhuang L. Wang D. Han J. Lam E. W. F. (2014). The FoxO-BNIP3 axis exerts a unique regulation of mTORC1 and cell survival under energy stress. Oncogene 33, 3183–3194. 10.1038/onc.2013.273 23851496 PMC4365448

[B92] Liu Z. Lenardo M. J. (2012). The role of LRRK2 in inflammatory bowel disease. Cell Res. 22, 1092–1094. 10.1038/cr.2012.42 22430149 PMC3391018

[B93] Liu Y. Baba Y. Ishimoto T. Tsutsuki H. Zhang T. Nomoto D. (2020). Fusobacterium nucleatum confers chemoresistance by modulating autophagy in oesophageal squamous cell carcinoma. Br. J. Cancer 124 (5), 963–974. 10.1038/s41416-020-01198-5 33299132 PMC7921654

[B94] Liu J. Qiu R. Liu R. Song P. Lin P. Chen H. (2022). Autophagy mediates Escherichia Coli-induced cellular inflammatory injury by regulating calcium mobilization, mitochondrial dysfunction, and endoplasmic reticulum stress. Int. J. Mol. Sci. 23, 14174. 10.3390/ijms232214174 36430657 PMC9698444

[B95] Lu G. Wu Z. Shang J. Xie Z. Chen C. zhang C. (2021). The effects of metformin on autophagy. Biomed. and Pharmacother. 137, 111286. 10.1016/J.BIOPHA.2021.111286 33524789

[B96] Lu J. Kornmann M. Traub B. (2023). Role of epithelial to mesenchymal transition in colorectal cancer. Int. J. Mol. Sci. 24, 14815. 10.3390/IJMS241914815 37834263 PMC10573312

[B97] Lucas C. Salesse L. Hanh M. Hoang T. Bonnet M. Sauvanet P. (2020). Autophagy of intestinal epithelial cells inhibits colorectal carcinogenesis induced by colibactin-producing *Escherichia coli* in ApcMin/+ mice. D. Mice 158, 1373–1388. 10.1053/j.gastro.2019.12.026 31917256

[B98] Luo Z. Wang H. Lin S. Liao L. Cai L. Zhang X. (2022). Study on the levels of N-nitrosamine compounds and untargeted metabolomics in patients with colorectal cancer. Anal. Bioanal. Chem. 414, 3483–3496. 10.1007/s00216-022-03969-w 35174409

[B99] Magistri P. Battistelli C. Strippoli R. Petrucciani N. Pellinen T. Rossi L. (2018). SMO inhibition modulates cellular plasticity and invasiveness in colorectal cancer. Front. Pharmacol. 8, 956. 10.3389/fphar.2017.00956 29456503 PMC5801594

[B100] Mäklin T. Taira A. Arredondo-Alonso S. Shao Y. Stratton M. R. Lawley T. D. (2024). Geographical variation in the incidence of colorectal cancer and urinary tract cancer is associated with population exposure to colibactin-producing *Escherichia coli* . Lancet Microbe 6, 101015. 10.1016/J.LANMIC.2024.101015 39644909

[B101] Marcucci F. Stassi G. De Maria R. (2016). Epithelial-mesenchymal transition: a new target in anticancer drug discovery. Nat. Rev. Drug Discov. 15, 311–325. 10.1038/nrd.2015.13 26822829

[B102] Mizushima N. Yoshimori T. Ohsumi Y. (2011). The role of atg proteins in autophagosome formation. Annu. Rev. Cell Dev. Biol. 27, 107–132. 10.1146/ANNUREV-CELLBIO-092910-154005 21801009

[B103] Moreira M. M. Carriço M. Capelas M. L. Pimenta N. Santos T. Ganhão-Arranhado S. (2024). The impact of pre-pro- and synbiotics supplementation in colorectal cancer treatment: a systematic review. Front. Oncol. 14, 1395966. 10.3389/fonc.2024.1395966 38807764 PMC11130488

[B104] Morgan E. Arnold M. Gini A. Lorenzoni V. Cabasag C. J. Laversanne M. (2023). Global burden of colorectal cancer in 2020 and 2040: incidence and mortality estimates from GLOBOCAN. Gut 72, 338–344. 10.1136/GUTJNL-2022-327736 36604116

[B105] Morrison D. J. Preston T. (2016). Formation of short chain fatty acids by the gut microbiota and their impact on human metabolism. Gut Microbes 7, 189–200. 10.1080/19490976.2015.1134082 26963409 PMC4939913

[B106] Mrkvicova A. Chmelarova M. Peterova E. Havelek R. Baranova I. Kazimirova P. (2019). The effect of sodium butyrate and cisplatin on expression of EMT markers. PLoS One 14, e0210889. 10.1371/journal.pone.0210889 30653577 PMC6336326

[B107] Munteanu C. Turnea M. A. Rotariu M. (2023). Hydrogen sulfide: an emerging regulator of oxidative stress and cellular Homeostasis-A comprehensive one-year review. Antioxidants (Basel) 12, 1737. 10.3390/ANTIOX12091737 37760041 PMC10526107

[B108] Nazio F. Bordi M. Cianfanelli V. Locatelli F. Cecconi F. (2019). Autophagy and cancer stem cells: molecular mechanisms and therapeutic applications. Cell Death Differ. 26, 690–702. 10.1038/s41418-019-0292-y 30728463 PMC6460398

[B109] Ni R. Jiang J. Zhao M. Huang S. Huang C. (2023). Knockdown of UBQLN1 functions as a strategy to inhibit CRC progression through the ERK-c-Myc pathway. Cancers (Basel) 15, 3088. 10.3390/cancers15123088 37370699 PMC10296216

[B110] Niklaus M. Adams O. Berezowska S. Zlobec I. Graber F. Slotta-Huspenina J. (2017). Expression analysis of LC3B and p62 indicates intact activated autophagy is associated with an unfavorable prognosis in Colon cancer. Oncotarget 8, 54604–54615. 10.18632/oncotarget.17554 28903368 PMC5589607

[B111] Park J. M. Huang S. Wu T. T. Foster N. R. Sinicrope F. A. (2013). Prognostic impact of beclin 1, p62/sequestosome 1 and LC3 protein expression in colon carcinomas from patients receiving 5-fluorouracil as adjuvant chemotherapy. Cancer Biol. Ther. 14, 100–107. 10.4161/cbt.22954 23192274 PMC3571991

[B112] Park C. H. Eun C. S. Han D. S. (2018). Intestinal microbiota, chronic inflammation, and colorectal cancer. Intest. Res. 16, 338–345. 10.5217/IR.2018.16.3.338 30090032 PMC6077304

[B113] Patil A. Singh N. Patwekar M. Patwekar F. Patil A. Gupta J. K. (2024). AI-driven insights into the microbiota: figuring out the mysterious world of the gut. Intell. Pharm. 3, 46–52. 10.1016/J.IPHA.2024.08.003

[B114] Perrott S. McDowell R. Murchie P. Cardwell C. Samuel L. (2021). SO-25 global rise in early-onset colorectal cancer: an association with antibiotic consumption? Ann. Oncol. 32, S213. 10.1016/j.annonc.2021.05.049

[B115] Poulogiannis G. McIntyre R. E. Dimitriadi M. Apps J. R. Wilson C. H. Ichimura K. (2010). PARK2 deletions occur frequently in sporadic colorectal cancer and accelerate Adenoma development in apc mutant mice. Proc. Natl. Acad. Sci. U. S. A. 107, 15145–15150. 10.1073/pnas.1009941107 20696900 PMC2930574

[B116] Pourali G. Kazemi D. Chadeganipour A. S. Arastonejad M. Kashani S. N. Pourali R. (2024). Microbiome as a biomarker and therapeutic target in pancreatic cancer. BMC Microbiol. 24 (1), 16–24. 10.1186/S12866-023-03166-4 38183010 PMC10768369

[B117] Pral L. P. Fachi J. L. Corrêa R. O. Colonna M. Vinolo M. A. R. (2021). Hypoxia and HIF-1 as key regulators of gut microbiota and host interactions. Trends Immunol. 42, 604–621. 10.1016/j.it.2021.05.004 34171295 PMC8283795

[B118] Procházková N. Falony G. Dragsted L. O. Licht T. R. Raes J. Roager H. M. (2023). Advancing human gut microbiota research by considering gut transit time. Gut 72, 180–191. 10.1136/gutjnl-2022-328166 36171079 PMC9763197

[B119] Qu S. Gao Y. Ma J. Yan Q. (2023). Microbiota-derived short-chain fatty acids functions in the biology of B lymphocytes: from differentiation to antibody formation. Biomed. and Pharmacother. 168, 115773. 10.1016/J.BIOPHA.2023.115773 39491858

[B120] Qureshi-Baig K. Kuhn D. Viry E. Pozdeev V. I. Schmitz M. Rodriguez F. (2020). Hypoxia-induced autophagy drives colorectal cancer initiation and progression by activating the PRKC/PKC-EZR (ezrin) pathway. Autophagy 16, 1436–1452. 10.1080/15548627.2019.1687213 31775562 PMC7469473

[B121] Rubinstein M. R. Wang X. Liu W. Hao Y. Cai G. Han Y. W. (2013). Fusobacterium nucleatum promotes colorectal carcinogenesis by modulating E-Cadherin/β-Catenin signaling *via* its FadA adhesin. Cell Host Microbe 14, 195–206. 10.1016/j.chom.2013.07.012 23954158 PMC3770529

[B122] Sah D. K. Arjunan A. Lee B. Jung Y. D. (2023). Reactive oxygen species and *H. pylori* infection: a comprehensive review of their roles in gastric cancer development. Antioxidants 12, 1712. 10.3390/ANTIOX12091712 37760015 PMC10525271

[B123] Sakitani K. Hirata Y. Hikiba Y. Hayakawa Y. Ihara S. Suzuki H. (2015). Inhibition of autophagy exerts anti-colon cancer effects *via* apoptosis induced by p53 activation and ER stress. BMC Cancer 15, 795. 10.1186/S12885-015-1789-5 26496833 PMC4620020

[B124] Salesse L. Lucas C. Hoang M. H. T. Sauvanet P. Rezard A. Rosenstiel P. (2021). Colibactin-producing Escherichia coli induce the formation of invasive carcinomas in a chronic inflammation-associated mouse model. Cancers (Basel) 13, 2060. 10.3390/cancers13092060 33923277 PMC8123153

[B125] Shang Y. Chen H. Ye J. Wei X. Liu S. Wang R. (2017). HIF-1α/Ascl2/miR-200b regulatory feedback circuit modulated the epithelial-mesenchymal transition (EMT) in colorectal cancer cells. Exp. Cell Res. 360, 243–256. 10.1016/j.yexcr.2017.09.014 28899657

[B126] Shao B. Z. Yao Y. Zhai J. S. Zhu J. H. Li J. P. Wu K. (2021). The role of autophagy in inflammatory bowel disease. Front. Physiol. 12, 621132. 10.3389/FPHYS.2021.621132 33633585 PMC7902040

[B127] Shen H. Yin L. Deng G. Guo C. Han Y. Li Y. (2018). Knockdown of Beclin-1 impairs epithelial-mesenchymal transition of colon cancer cells. J. Cell Biochem. 119, 7022–7031. 10.1002/jcb.26912 29738069

[B128] Silva-García O. Valdez-Alarcón J. J. Baizabal-Aguirre V. M. (2019). Wnt/β-catenin signaling as a molecular target by pathogenic bacteria. Front. Immunol. 10, 2135. 10.3389/fimmu.2019.02135 31611869 PMC6776594

[B129] Singh S. B. Carroll-Portillo A. Lin H. C. (2023). Desulfovibrio in the gut: the enemy within? Microorganisms 11, 1772. 10.3390/MICROORGANISMS11071772 37512944 PMC10383351

[B130] Singhal R. Shah Y. M. (2020). Oxygen battle in the gut: hypoxia and hypoxia-inducible factors in metabolic and inflammatory responses in the intestine. J. Biol. Chem. 295, 10493–10505. 10.1074/jbc.REV120.011188 32503843 PMC7383395

[B131] Sittipo P. Lobionda S. Choi K. Sari I. N. Kwon H. Y. Lee Y. K. (2018). Toll-like receptor 2-Mediated suppression of colorectal cancer pathogenesis by polysaccharide A from Bacteroides fragilis. Front. Microbiol. 9, 1588. 10.3389/FMICB.2018.01588 30065713 PMC6056687

[B132] Stidham R. W. Higgins P. D. R. (2018). Colorectal cancer in inflammatory bowel disease. Clin. Colon Rectal Surg. 31, 168–178. 10.1055/S-0037-1602237 29720903 PMC5929884

[B133] Su W. Chen Y. Cao P. Chen Y. Guo Y. Wang S. (2020). Fusobacterium nucleatum promotes the development of ulcerative colitis by inducing the autophagic cell death of intestinal epithelial. Front. Cell Infect. Microbiol. 10, 594806. 10.3389/fcimb.2020.594806 33330137 PMC7728699

[B134] Su Y. Fan X. Cai X. Ning J. Shen M. (2024). Effects of fecal microbiota transplantation combined with selenium on intestinal microbiota in mice with colorectal cancer. Biochem. Biophys. Res. Commun. 733, 150580. 10.1016/J.BBRC.2024.150580 39213702

[B135] Sun X. Zhu M. J. (2017). AMP-activated protein kinase: a therapeutic target in intestinal diseases. Open Biol. 7, 170104. 10.1098/RSOB.170104 28835570 PMC5577448

[B136] Takiishi T. Fenero C. I. M. Câmara N. O. S. (2017). Intestinal barrier and gut microbiota: shaping our immune responses throughout life. Tissue Barriers 5, e1373208. 10.1080/21688370.2017.1373208 28956703 PMC5788425

[B137] Tito R. Y. Verbandt S. Aguirre Vazquez M. Lahti L. Verspecht C. Lloréns-Rico V. (2024). Microbiome confounders and quantitative profiling challenge predicted microbial targets in colorectal cancer development. Nat. Med. 30 (5), 1339–1348. 10.1038/s41591-024-02963-2 38689063 PMC11108775

[B138] Tran S. Juliani J. Harris T. J. Evangelista M. Ratcliffe J. Ellis S. L. (2024). BECLIN1 is essential for intestinal homeostasis involving autophagy-independent mechanisms through its function in endocytic trafficking. Commun. Biol. 7 (1), 209–213. 10.1038/s42003-024-05890-7 38378743 PMC10879175

[B139] Tzeng A. Sangwan N. Jia M. Liu C. C. Keslar K. S. Downs-Kelly E. (2021). Human breast microbiome correlates with prognostic features and immunological signatures in breast cancer. Genome Med. 13, 60. 10.1186/S13073-021-00874-2 33863341 PMC8052771

[B140] van Vorstenbosch R. Cheng H. R. Jonkers D. Penders J. Schoon E. Masclee A. (2023). Systematic review: contribution of the gut microbiome to the volatile metabolic fingerprint of colorectal neoplasia. Metabolites 13, 55. 10.3390/metabo13010055 PMC9865897 36676980

[B141] Vancamelbeke M. Vermeire S. (2017). The intestinal barrier: a fundamental role in health and disease. Expert Rev. Gastroenterol. Hepatol. 11, 821–834. 10.1080/17474124.2017.1343143 28650209 PMC6104804

[B142] Vescovo T. Pagni B. Piacentini M. Fimia G. M. Antonioli M. (2020). Regulation of autophagy in cells infected with oncogenic human viruses and its impact on cancer development. Front. Cell Dev. Biol. 8, 47. 10.3389/fcell.2020.00047 32181249 PMC7059124

[B143] Walter L. Canup B. Pujada A. Bui T. A. Arbasi B. Laroui H. (2020). Matrix metalloproteinase 9 (MMP9) limits reactive oxygen species (ROS) accumulation and DNA damage in colitis-associated cancer. Cell Death Dis. 11, 767. 10.1038/s41419-020-02959-z 32943603 PMC7498454

[B144] Wang Y. Wu N. Pang B. Tong D. Sun D. Sun H. (2017). TRIB1 promotes colorectal cancer cell migration and invasion through activation MMP-2 *via* FAK/Src and ERK pathways. Oncotarget 8, 47931–47942. 10.18632/oncotarget.18201 28624785 PMC5564616

[B145] Wang J. Gu X. Yang J. Wei Y. Zhao Y. (2019). Gut microbiota dysbiosis and increased plasma LPS and TMAO levels in patients with preeclampsia. Front. Cell Infect. Microbiol. 9, 409. 10.3389/FCIMB.2019.00409 31850241 PMC6901393

[B146] Wang Y. Xu X. Marshall J. E. Gong M. Zhao Y. Dua K. (2021). Loss of hyaluronan and proteoglycan link Protein-1 induces tumorigenesis in colorectal cancer. Front. Oncol. 11, 754240. 10.3389/fonc.2021.754240 34966673 PMC8710468

[B147] Wang Q. Hu T. Zhang Q. Zhang Y. Dong X. Jin Y. (2025). Fusobacterium nucleatum promotes colorectal cancer through neogenesis of tumor stem cells. J. Clin. Invest 135, e181595. 10.1172/JCI181595 39656543 PMC11785920

[B148] Wei L. Q. Cheong I. H. Yang G. H. Li X. G. Kozlakidis Z. Ding L. (2021). The application of high-throughput technologies for the study of microbiome and cancer. Front. Genet. 12, 699793. 10.3389/fgene.2021.699793 34394190 PMC8355622

[B149] Wilson M. R. Jiang Y. Villalta P. W. Stornetta A. Boudreau P. D. Carrá A. (2019). The human gut bacterial genotoxin colibactin alkylates DNA. Sci. (1979) 363, eaar7785. 10.1126/science.aar7785 PMC6407708 30765538

[B150] Wu Z. Q. Brabletz T. Fearon E. Willis A. L. Hu C. Y. Li X. Y. (2012). Canonical wnt suppressor, Axin2, promotes colon carcinoma oncogenic activity. Proc. Natl. Acad. Sci. U. S. A. 109, 11312–11317. 10.1073/pnas.1203015109 22745173 PMC3396472

[B151] Wu S. Shen Y. Zhang S. Xiao Y. Shi S. (2020). Salmonella interacts with autophagy to offense or defense. Front. Microbiol. 11, 721. 10.3389/FMICB.2020.00721 32390979 PMC7188831

[B152] Wu J. N. Lin L. Luo S. B. Qiu X. Z. Zhu L. Y. Chen D. (2021a). SphK1-driven autophagy potentiates focal adhesion paxillin-mediated metastasis in colorectal cancer. Cancer Med. 10, 6010–6021. 10.1002/CAM4.4129 34268882 PMC8419751

[B153] Wu N. Jiang M. Liu H. Chu Y. Wang D. Cao J. (2021b). LINC00941 promotes CRC metastasis through preventing SMAD4 protein degradation and activating the TGF-β/SMAD2/3 signaling pathway. Cell Death Differ. 28, 219–232. 10.1038/s41418-020-0596-y 32737443 PMC7853066

[B154] Wu R. Zhang Y. Xu X. You Q. Yu C. Wang W. (2023). Exosomal B7-H3 facilitates colorectal cancer angiogenesis and metastasis through AKT1/mTOR/VEGFA pathway. Cell Signal 109, 110737. 10.1016/j.cellsig.2023.110737 37263461

[B155] Xi Y. Xu P. (2021). Global colorectal cancer burden in 2020 and projections to 2040. Transl. Oncol. 14, 101174. 10.1016/j.tranon.2021.101174 34243011 PMC8273208

[B156] Xiao T. Zhu W. Huang W. Lu S. S. Li X. H. Xiao Z. Q. (2018). RACK1 promotes tumorigenicity of colon cancer by inducing cell autophagy. Cell Death Dis. 9, 1148. 10.1038/S41419-018-1113-9 30451832 PMC6242835

[B157] Xu Z. Zhu C. Chen C. Zong Y. Feng H. Liu D. (2018). CCL19 suppresses angiogenesis through promoting miR-206 and inhibiting Met/ERK/Elk-1/HIF-1α/VEGF-A pathway in colorectal cancer. Cell Death Dis. 9, 974. 10.1038/s41419-018-1010-2 30250188 PMC6155262

[B158] Xu W. Hua Z. Wang Y. Tang W. Ou W. Liu F. (2024). AMBRA1 promotes intestinal inflammation by antagonizing PP4R1/PP4c mediated IKK dephosphorylation in an autophagy-independent manner. Cell Death and Differ. 31 (5), 618–634. 10.1038/s41418-024-01275-9 PMC11094188 38424148

[B159] Xue Y. Zhu M. J. (2018). Suppressing autophagy: a strategy by *Escherichia coli* O157:H7 for its survival on host epithelial cells. Cell Death Dis. 9, 64. 10.1038/S41419-017-0095-3 29352117 PMC5833748

[B160] Xue W. Yang L. Chen C. Ashrafizadeh M. Tian Y. Sun R. (2024). Wnt/β-catenin-driven EMT regulation in human cancers. Cell. Mol. Life Sci. 81 (1), 79–19. 10.1007/S00018-023-05099-7 38334836 PMC10857981

[B161] Yang M. Zhao H. Guo L. Zhang Q. Zhao L. Bai S. (2015). Autophagy-based survival prognosis in human colorectal carcinoma. Oncotarget 6, 7084–7103. 10.18632/oncotarget.3054 25762626 PMC4466671

[B162] Yang L. Liu C. Zhao W. He C. Ding J. Dai R. (2018). Impaired autophagy in intestinal epithelial cells alters gut microbiota and host immune responses. Appl. Environ. Microbiol. 84, e00880-18. 10.1128/AEM.00880-18 30006408 PMC6121970

[B163] Yu L. C. H. (2018). Microbiota dysbiosis and barrier dysfunction in inflammatory bowel disease and colorectal cancers: exploring a common ground hypothesis. J. Biomed. Sci. 25, 79. 10.1186/S12929-018-0483-8 30413188 PMC6234774

[B164] Yu T. C. Guo F. Yu Y. Sun T. Ma D. Han J. (2017). Fusobacterium nucleatum promotes chemoresistance to colorectal cancer by modulating autophagy. Cell 170, 548–563. 10.1016/j.cell.2017.07.008 28753429 PMC5767127

[B165] Yu M. R. Kim H. J. Park H. R. (2020). Fusobacterium nucleatum accelerates the progression of colitis-associated colorectal cancer by promoting emt. Cancers (Basel) 12, 2728–19. 10.3390/CANCERS12102728 32977534 PMC7598280

[B166] Yu H. Li X. X. Han X. Chen B. X. Zhang X. H. Gao S. (2023). Fecal microbiota transplantation inhibits colorectal cancer progression: reversing intestinal microbial dysbiosis to enhance anti-cancer immune responses. Front. Microbiol. 14, 1126808. 10.3389/fmicb.2023.1126808 37143538 PMC10151806

[B167] YuJ.FengL.LuoZ.YangJ.ZhangQ.LiuC. (2024). Interleukin-10 deficiency suppresses colorectal cancer metastasis by enriching gut Parabacteroides distasonis. J. Adv. Res. 10.1016/J.JARE.2024.11.024 39571733

[B168] Yu Y. Yin H. Wu B. Zhao W. Wang Y. Aili A. (2025). Fusobacterium nucleatum promotes colorectal cancer liver metastasis *via* miR-5692a/IL-8 axis by inducing epithelial-mesenchymal transition. J. Biomed. Sci. 32, 5–15. 10.1186/s12929-024-01097-4 39757156 PMC11702224

[B169] Zeng H. Umar S. Rust B. Lazarova D. Bordonaro M. (2019). Secondary bile acids and short chain fatty acids in the colon: a focus on colonic microbiome, cell proliferation, inflammation, and cancer. Int. J. Mol. Sci. 20, 1214. 10.3390/ijms20051214 30862015 PMC6429521

[B170] Zhang J. Chu D. Kawamura T. Tanaka K. He S. (2019). GRIM-19 repressed hypoxia-induced invasion and EMT of colorectal cancer by repressing autophagy through inactivation of STAT3/HIF-1α signaling axis. J. Cell Physiol. 234, 12800–12808. 10.1002/jcp.27914 30537081 PMC13483207

[B171] Zhang N. Kandalai S. Zhou X. Hossain F. Zheng Q. (2023). Applying multi-omics toward tumor microbiome research. iMeta 2, e73. 10.1002/IMT2.73 38868335 PMC10989946

[B172] Zhang X. Li B. Lan T. Chiari C. Ye X. Wang K. (2024). The role of interleukin-17 in inflammation-related cancers. Front. Immunol. 15, 1479505. 10.3389/fimmu.2024.1479505 39906741 PMC11790576

[B173] Zhang H. Tian Y. Xu C. Chen M. Xiang Z. Gu L. (2025). Crosstalk between gut microbiotas and fatty acid metabolism in colorectal cancer. Cell Death Discov. 11 (1), 78–13. 10.1038/s41420-025-02364-5 40011436 PMC11865559

[B174] Zhao L. Y. Mei J. X. Yu G. Lei L. Zhang W. H. Liu K. (2023). Role of the gut microbiota in anticancer therapy: from molecular mechanisms to clinical applications. Signal Transduct. Target. Ther. 8 (1), 201–227. 10.1038/s41392-023-01406-7 37179402 PMC10183032

[B175] Zheng C. G. Chen R. Xie J. B. Liu C. B. Jin Z. Jin C. (2015). Immunohistochemical expression of Notch1, Jagged1, NF-κB and MMP-9 in colorectal cancer patients and the relationship to clinicopathological parameters. Cancer Biomarkers 15, 889–897. 10.3233/CBM-150533 26406415 PMC12965476

[B176] Zhu Y. Huang S. Chen S. Chen J. Wang Z. Wang Y. (2021). SOX2 promotes chemoresistance, cancer stem cells properties, and epithelial-mesenchymal transition by β-catenin and Beclin1/autophagy signaling in colorectal cancer. Cell Death Dis. 12, 449. 10.1038/S41419-021-03733-5 33953166 PMC8100126

[B177] Ziegler P. K. Bollrath J. Pallangyo C. K. Matsutani T. Canli Ö. De Oliveira T. (2018). Mitophagy in intestinal epithelial cells triggers adaptive immunity during tumorigenesis. Cell 174, 88–101. 10.1016/j.cell.2018.05.028 29909986 PMC6354256

